# Populations of the Parasitic Plant *Phelipanche ramosa* Influence Their Seed Microbiota

**DOI:** 10.3389/fpls.2020.01075

**Published:** 2020-07-17

**Authors:** Sarah Huet, Jean-Bernard Pouvreau, Erwan Delage, Sabine Delgrange, Coralie Marais, Muriel Bahut, Philippe Delavault, Philippe Simier, Lucie Poulin

**Affiliations:** ^1^ Laboratoire de Biologie et Pathologie Végétales, EA 1157, SFR 4207 QUASAV, UFR Sciences et Techniques, Université de Nantes, Nantes, France; ^2^ Laboratoire des Sciences du Numérique de Nantes, UMR CNRS 6004, IMT Atlantique, ECN, Université de Nantes, Nantes, France; ^3^ Plateau Technique Mutualisé ANAN, SFR 4207 QUASAV, Beaucouzé, France

**Keywords:** parasitic weeds, seed germination, host adaptation, seed microbiota, core microbiota

## Abstract

Seeds of the parasitic weed *Phelipanche ramosa* are well adapted to their hosts because they germinate and form haustorial structures to connect to roots in response to diverse host-derived molecular signals. *P. ramosa* presents different genetic groups that are preferentially adapted to certain hosts. Since there are indications that microbes play a role in the interaction especially in the early stages of the interaction, we studied the microbial diversity harbored by the parasitic seeds with respect to their host and genetic group. Twenty-six seed lots from seven cropping plots of three different hosts—oilseed rape, tobacco, and hemp—in the west of France were characterized for their bacterial and fungal communities using 16S rRNA gene and ITS (Internal transcribed spacer) sequences, respectively. First seeds were characterized genetically using twenty microsatellite markers and phenotyped for their sensibility to various germination stimulants including strigolactones and isothiocyanates. This led to the distinction of three *P. ramosa* groups that corresponded to their host of origin. The observed seed diversity was correlated to the host specialization and germination stimulant sensitivity within *P. ramosa* species. Microbial communities were both clustered by host and plot of origin. The seed core microbiota was composed of seventeen species that were also retrieved from soil and was in lower abundances for bacteria and similar abundances for fungi compared to seeds. The host-related core microbiota of parasitic seeds was limited and presumably well adapted to the interaction with its hosts. Two microbial candidates of *Sphingobacterium* species and *Leptosphaeria maculans* were especially identified in seeds from oilseed rape plots, suggesting their involvement in host recognition and specialization as well as seed fitness for *P. ramosa* by improving the production of isothiocyanates from glucosinolates in the rhizosphere of oilseed rape.

## Introduction

Branched broomrape, *Phelipanche ramosa*, is a root holoparasitic plant belonging to the *Orobanchaceae* family. It is the most widespread parasitic weed in intensive crop cultivated systems in the Mediterranean region and can parasitizes various host crops including oilseed rape (*Brassica napus*), hemp (*Cannabis sativa*), tomato (*Solanum lycopersicum*), tobacco (*Nicotiana tabacum*), sunflower (*Helianthus annuus*), and melon (*Cucumis melo*) resulting in significant yield and economical losses (Tsialtas and Eleftherohorinos, 2011; Parker, 2012). Because *P. ramosa* is an achlorophyllous plant, it relies entirely on its host for nutrition thanks to haustoria that directly connect to host root phloem tissues (Westwood, 2013)⁠. The ecological fitness of this parasite is maximized by a small seed size, a large seed bank produced per plant and a long survival of seeds that remain viable in dormancy in the soil for decades (Castejon et al., 1991; Fleischmann et al., 1995; Joel et al., 2007)⁠. Furthermore, the seeds possess an inducible and fine adapted perception of host plant-related allelochemical signals for initiating germination and haustorium formation, conferring parasite an optimized trait of life.

Indeed, seeds only germinate in response to metabolites that are exuded by their host plant roots. The first generic germination stimulants (GS) discovered are canonical and non-canonical strigolactones (Yoneyama et al., 2010; Screpanti et al., 2016) which are phytohormones that are commonly found in rhizosphere of many plants (Delaux et al., 2012)⁠. Various *KAI2/HTL* paralogs (KARRIKIN INSENSITIVE2 - HYPOSENSITIVE TO LIGHT) code for strigolactone receptors in parasitic plants due to gene duplication and diversification within Orobanchaceae family (Conn et al., 2015; Toh et al., 2015)⁠. Other GS described specifically in *Brassicaceae* rhizosphere are the isothiocyanates (ITC), the products of glucosinolates hydrolysis by myrosinase enzymes from plant or microbial origin (Auger et al., 2012). Yet, no receptor has been described in parasitic plants for ITC. To connect to their host roots, germinating seeds elongate a radicle and form appendices, a.k.a haustorium, in response to other rhizosphere signals including cytokinin-related compounds (Goyet et al., 2017; Goyet et al., 2019).

*P. ramosa* seeds recognize and interact with their hosts differently depending on their own genetic structure. A recent study showed that at least three main genotypic groups define *P. ramosa* genetic diversity in European *P. ramosa* populations from the Mediterranean basin (Stojanova et al., 2019)⁠. The genetic diversity patterns are attributed to a combination of both geographic provenance and host of origin. Those genetic groups show a preference for specific hosts even though interactions were not exclusive. Indeed, genetic group 1 was mostly associated with winter oilseed rape while genetic group 2a was associated to hemp and winter oilseed rape, and genetic group 2b was more diversified and associated to various crops but mostly tobacco.

So far, host specialization has only been studied from the point of view of the plant-plant interaction and omitting potential microbial implications (Stojanova et al., 2019)⁠. However, microbiota may have a key role in the interaction and possibly in the geographic and host co-specialization. One *in vitro* study showed a global microbiome transfer between the *Phelipanche aegyptiaca* parasitic plant and the tomato host plant after parasitic attachment. And one of the tomato root endophytic strains was shown to inhibit parasitic attachment to roots in experimental conditions (Iasur Kruh et al., 2017)⁠. Another *in situ* study on *Orobanche hederae* vs ivy showed that root and shoot parasitic plant microbiota were derived from host plant microbiota (Fitzpatrick and Schneider, 2020)⁠. In addition, arbuscular mycorrhizal fungi and *Rhizobium* bacteria were also proposed as biocontrol organisms as they both reduce the parasitism on *Orobanche cumana* and *Orobanche crenata* respectively (Mabrouk et al., 2007; Louarn et al., 2012)⁠. However, the role of microbiota in the early stages of the parasitic cycle has not been examined although there are several indications that microbiota could interact with GS, GS precursors, haustorium-inducing factor and inhibitors (Masteling et al., 2019)⁠. For instance, while many microorganisms are sensitive to ITC which are antimicrobial sulfur metabolite (Smith and Kirkegaard, 2002; Romeo et al., 2018)⁠ some have acquired a certain tolerance to ITC (Welte et al., 2016)⁠ and some exhibit a myrosinase activity to out-compete the ITC sensitive microorganisms (Ohtsuru et al., 1969; Albaser et al., 2016)⁠. Even some ITC resistant microorganisms hydrolyze glucosinolates for a sugar uptake (Szűcs et al., 2018)⁠. Moreover, strigolactones were also described as a rhizosphere signal for mycorrhizal fungi recruitment and are thus largely involved in the plant—microorganisms interactions (Besserer et al., 2006).

Parasitic seeds may also harbor microbial communities that have a functional effect in the early steps of seed conditioning and germination (Shade et al., 2017)⁠. Those communities can be transmitted vertically during the parasitic plant development and life cycle, but also horizontally through environmental transfer *via* pollinator, atmospheric or soil contamination (Nelson, 2018). As described in several seed—microbe associations, some seed endophytes that are recruited and selected have a great potential for the seed fitness (Cankar et al., 2005; Puente et al., 2009; Hubbard et al., 2012)⁠ and some are directly involved in seed metabolisms (Goggin et al., 2015)⁠.

As microbes can interact with the parasitic plants, notably during the early stages of the interaction, they may have played a role in specialization. Thus, in this study, we analyzed the microbiota carried by parasitic seed according to their host and population of origin. We first characterized genetically the sampled parasitic seeds using single sequence repeats markers (SSR) and phenotypically by testing a range of GS reflecting the synthetic and natural diversity. The study of the microbiota associated with the parasitic seeds led to infer factors structuring microbial composition and allowed to decipher the global and host core seed microbiota that were selected. Some core microbial candidates were particularly highlighted as being able to breakdown or promote GS suggesting that they may have been involved in the interaction and the specialization of seeds with their hosts.

## Material and Methods

### Seed Lots

**Figure 1:**
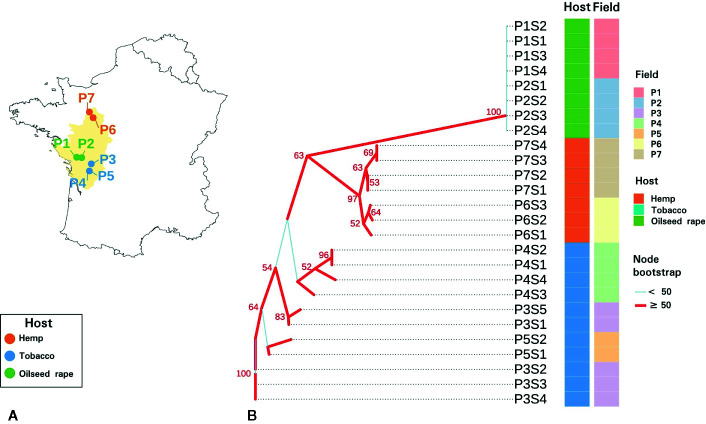
Sampling and genetic characteristics of seed samples. **(A)** Map of the seven sampled *P. ramosa* plots in western France. **(B)** Neighbor joining phylogenetic tree of parasitic seeds lots based on Bruvo genetic distances measured on sequence repeats marker (SSR) triplicate consensuses. Plots and hosts of sampling are indicated in column. Branches with a bootstrap higher than 50 are indicated in red while branches with a bootstrap inferior than 50 are indicated in light blue.

Twenty-six seed lots were harvested in seven different plots in the west of France at fruiting stage (**Figure 1**). Oilseed rape broomrape was prospected in the heavily infested region Poitou-Charentes in the early July 2017. Tobacco broomrape was collected in Poitou-Charentes early September 2017 while hemp broomrape was prospected in the Sarthe department in mid-September 2017. At each plot location, 3 to 4 spots of about 5–20 m^2^ - depending on the parasite coverage - of broomrape mature stalks were collected (**Figure S1**). At each sampling spot, superficial soil (0–20 cm in depth) was also collected. Informations on plots and crop rotation history are available in **Tables S1** and **S2**. Floral scapes with capsules were placed in a closed container that was manually shacked for several minutes. To get rid of dust and plant debris, raw samples were sieved at different grain sizes in 3 fractions of <180 µm, 180–250 µm, and >250 µm. Particles bigger than 250 µm and smaller than 180 were discarded and only particles of 150 to 250 µm were collected, which correspond to the seed fraction without debris. After sieving, the seed lots were conserved in glass jars in a dry culture chamber at 25°C and kept in the dark. The two reference batches of seeds that were also used, Pram120 (*Brassica napus*, Saint Jean d’Angély, 2015) and Pram121 (*Canabis* sativa, Ossey-les-Trois-Maisons, 2017), were kindly provided by C. Jestin (Terres Inovia).

### Seed Genotyping

DNA was extracted in triplicates with the NucleoSpin^®^ Plant II kit from MACHEREY-NAGEL and diluted at 20 ng/µl for further microsatellite SSR characterization using routine primers (Stojanova et al., 2019)⁠. SSR genotyping was performed at the GENTYANE platform (http://gentyane.clermont.inra.fr/). From the obtained FASTA files, amplicon sizes were derived for the 20 SSR markers of the 26 seed samples using the R software version 3.5.2 (2018-12-20) and the package Fragman (1.0.9) (Covarrubias-Pazaran et al., 2016)⁠. The SSR data were then analyzed using the poppr package (2.8.2) (Kamvar et al., 2014)⁠. Unique MultiLocus Genotypes (MLGs), defined as a unique combination of alleles, were associated to each seed lot and unrooted Neighbor-Joining tree was plotted using Bruvo genetic distance accounting for microsatellite evolution with 9,999 bootstrap replicates (Bruvo et al., 2004). In order to anchor the samples with reference lab lots, Bruvo genetic distance between Pram120, the laboratory reference lot of genetic group 1, and all samples was calculated and scored as a genetic factor.

### Seed Germination Phenotyping in Responses to GS Standard Molecules

Seed germination assays were performed on sterile seeds in 96-well plates according to (Pouvreau et al., 2013)⁠. GS molecules were tested in triplicates over a 10 fold serial concentration range from 10^-13^ to 10^-6^ M (0.1% acetonitrile; final volume 100 µl). The tested GS were: (+)-GR24, (-)-GR24, (+)-2*’-epi*-GR24, (-)-2*’-epi*-GR24, (±)-GR24 (racemic mix of (+)-GR24 and (-)-GR24) and 2-PEITC (2-phenylethyl isothiocyanate). The use of four stereoisomers of GR24, in addition to the usual mix of racGR24, allowed testing a diversity of pure strigolactones. Also it permitted to mimic the conformations of (i) known natural strigolactones a.k.a (+)-GR24 and (-)-2’-*epi*-GR24 with identical stereochemistry as 5-deoxystrigol and 4-deoxyorobanchol respectively; (ii) an unknown KAI2-Ligand (KL): (-)-GR24 and (iii) a non natural compound: (+)-2’-*epi*-GR24 (Scaffidi et al., 2014; Conn et al., 2015; Flematti et al., 2016; de Saint Germain et al., 2019; Machin et al., 2020). Strigolactones molecules were kindly provided by F-D Boyer (Centre National de la Recherche Scientifique,[CNRS]-INRAE Gif-sur-Yvette, France). A log logistic regression was modelled using the drm function the Drc (3.0-1) package and the EC50 (Half maximal Effective Concentration) and the standard deviation (Sd) were determined from the model (Ritz et al., 2015)⁠. These two variables were then tested by an ANOVA with a Tukey post-hoc (p-value < 0.05). GS sensibility EC50 were plotted against the phylogenetic tree and history of sensitivity traits was inferred using the phytool package (Revell, 2012)⁠.

### Amplicon Library Construction and MiSeq Sequencing

For each seed sample, three biological replicates of four hundred mg of seeds were macerated in 25 ml of PBS 1% and Tween 0,05% in a 50 ml falcon tube for 150 min at 400 rpm to obtain microbial suspensions. Seeds were discarded and macerates were centrifuged for 15 min at 6000 g and resuspended in 2 ml of solution. DNA extraction was carried out from 200 µl of concentrated microbial suspension using the NucleoSpin ^®^ Soil kit (Macherey-Nagel) according to manufacturer instructions for the 26 samples. Negative controls with only PBS and water and a positive control with a 10 bacteria mock (Barret et al., 2015) were also processed. For soil samples, three hundred milligrams were processed in triplicates for DNA extraction by the NucleoSpin ^®^ Soil kit. Then, two-round PCRs were carried out to construct the amplicon libraries. Two taxonomic markers were amplified, V4 region of 16S rRNA and ITS (internal transcribed spacer‐1), using respectively primers 16S_515f (GTGCCAGCMGCCGCGGTAA) and 16S_806r (GGACTACVSGGGTATCTAAT) (Caporaso et al., 2011)⁠ and ITS1_f (CTTGGTCATTTAGAGGAAGTAA) and ITS2 (GCTGCGTTCTTCATCGATGC) (Buée et al., 2009)⁠. The utilized forward and reverse primers carried the 5′-CTTTCCCTACACGACGCTCTTCCGATCT-3′ and 5′-GGAGTTCAGACGTGTGCTCTTCCGATCT-3′ tails, respectively. The first PCR was carried out with AccuPrimeTM Taq DNA Polymerase High Fidelity (REF 12346-086, Invitrogen by Thermo Fisher Scientific). The PCR cycle conditions were an initial denaturation of 94°C for 3 min followed by 35 cycles of denaturation at 94°C for 30 s, primer annealing at 50°C for 45 s and extension at 68°C for 90 s plus a final extension at 68°C for 10 min. Afterwards, amplicons were purified using Sera-Mag™ Magnetic carboxylate modified particles (GE Healthcare, 24152105050250). Then, a second PCR amplification was performed to incorporate Illumina adapters and barcodes using GoTaq^®^ G2 DNA Polymerase (REF M7845, Promega). The second PCR conditions were 94°C for 60 s followed by 12 cycles of 94° for 60 s, 55°C for 60 s and 72°C for 60 s and a final extension step of 72°C for 10 min. Subsequently, amplicons were purified as for the first PCR. Thereafter, amplicons were quantified using Quant-iT™ PicoGreen™ dsDNA Assay kit (Invitrogen P11496) and an equimolar pool was dosed by qPCR using KAPA Library Quant Illumina kit (Roche, 07960140001). The equimolar pool added with 5% of phiX phage mix was finally diluted to 12 pM and 600 µl was added in the sequencer cartridge (MiSeq Reagent Kit v3 MS-102-3003). Library constructions and MiSeq sequencing were performed at the ANAN platform (SFR QUASAV, INRAE Beaucouzé).

### Microbial Community Bioinformatic Analysis

Raw reads were processed using microSysMics (https://bio.tools/microSysMics), a workflow that relies on the Qiime2 toolbox (Bolyen et al., 2019)⁠. Reads quality was checked using Fastqc^1^ and Multiqc (Ewels et al., 2016)⁠. Illumina adaptators were removed, reads were filtered and trimmed using a home made script exploiting Cutadapt (Martin, 2011)⁠. Denoising was implemented using Dada2 which discards chimeric and erroneous sequences, retrieves individual true biological sequences called Amplicon Sequence Variants (ASV) and computes their frequency (Callahan et al., 2016)⁠. Taxonomy was assigned based on the 16S rRNA gene Silva database (Quast et al., 2012) and on the UNITE ITS database (Nilsson et al., 2019)⁠. Once the abundance table and the taxonomic table were obtained, subsequent analyses were implemented on R studio using Phyloseq package (1.24.2). To get rid of possible reagent contaminants (Salter et al., 2014)⁠, we identified and removed contaminant ASV using iscontaminant function of the Decontam package (1.1.2) with the “either” method and a threshold at 0.1 (Davis et al., 2018).

Different ecological indices were used to estimate alpha-diversity: (i) the observed richness that correspond to the number of detected ASVs and (ii) the Shannon and (iii) inverse Simpson’s index. The indices were calculated on rarefied dataset: rarefaction threshold was set at 1,500 and 2,000 reads per sample for 16S and ITS datasets, respectively. Rarefaction removed 56 and 57 ASVs from 16S and ITS datasets, respectively. Differences in richness and alpha-diversity were evaluated as whole by an ANOVA test with *post hoc* Tuckey test between each variable. Beta diversity was investigated by Bray-Curtis ordination (Bray and Curtis, 1957; Beals, 1984). Bray-Curtis distances were calculated on normalized ASV abundance i.e. ASV counts were divided by the number of reads per sample and multiplied by 10^6^. Afterwards, distance matrices were ordinated using a Principal Coordinate Analysis (PCoA).

To assess the weight of each variable on the dissimilarity, distance-based redundancy analysis were performed (capscale function in vegan package 2.5-4) with *post hoc* permutation test for redundancy analysis (anova.cca function in vegan package 2.5-4 with 999 permutations) to extract the model and the residues sum of square allowing us to calculate their mean of square and the adjusted r2 (function RsquareAdj in vegan package 2.5-4) for each model.

Taxonomic composition analysis was conveyed by the Phyloseq package and the UpSetR package (1.3.3). Sequences of unidentified ASV were compared with the NCBI database using BLAST (Altschul et al., 1990).

## Results

### Genotypes Representing *P. ramosa* Diversity in France

SSR amplicon sizes were relatively homogeneous among triplicates and 89.4% of the samples showed a standard deviation (sd) under 0.5 nucleotide and the maximal sd was 1.73. On the slightly diverging triplicates, consensus sizes were selected on the majority value (2 out of 3 triplicates). SSR amplicon size consensuses from the triplicates were reported in **Table S3**. The neighbor joining phylogenetic tree based on the 20 SSR markers revealed two distinct monophyletic groups (**Figure 1**). Parasitic seed samples originating from oilseed rape plots formed a monophyletic group with a bootstrap of 100%. This group corresponded to the genetic group 1 described for *P. ramosa* as Pram120, a lab reference seed lot clustered with this clade (data not shown). Seeds originating from hemp plots also clustered in a monophyletic group supported by a node bootstrap of 97% and clustered with Pram121, a lab seed lot of genetic group 2a (data not shown) (Stojanova et al., 2019)⁠. Seeds from tobacco plots presented a paraphyletic group. Seeds of genetic group 1 were genetically more distant to seeds originating from tobacco and hemp plots. Also, regarding intra-group diversity, heterozygosity (He) was higher for tobacco-related seeds (0.2240) than for hemp (0.0951) and oilseed rape (0.0617) – related parasitic seeds.

### Seed Sensitivity to GS According to Their Originating Host

**Figure 2:**
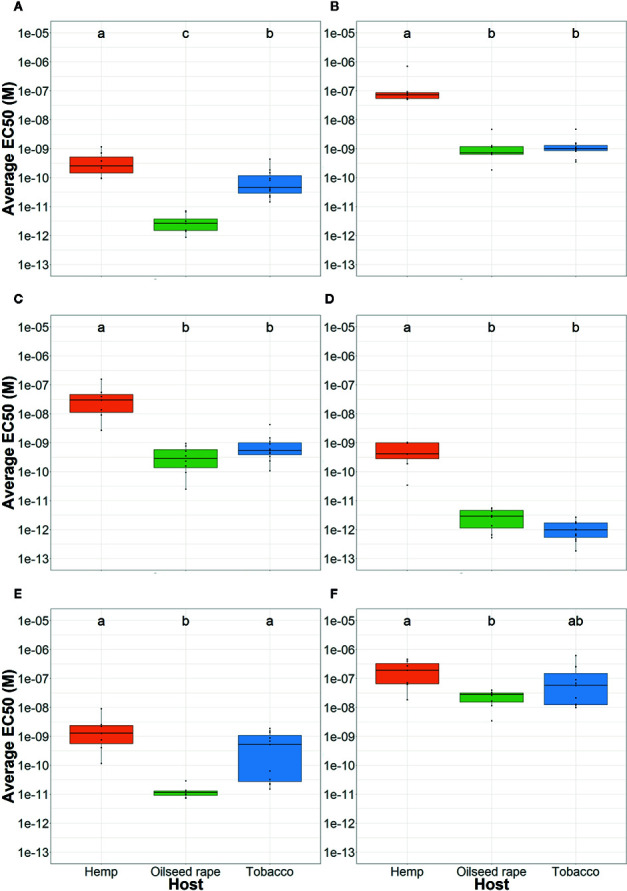
Average *P. ramosa* seed sensitivity per sampled host of origin. This was measured as the average EC50 per host group (orange: hemp, green: oilseed rape, blue: tobacco) in response to **(A)** (+)-GR24, **(B)** (-)-GR24, **(C)** (+)-2’-*epi*-GR24, **(D)** (-)-2’-*epi*-GR24, **(E)** (±)-GR24, and **(F)** 2-PEITC. For each seed sample, germination stimulant (GS) molecules were tested in triplicates. Different lowercase letters represent statistically different groups (Anova with post-hoc Tukey, p-value < 0.05).

All tested GS induced a maximum germination rate between 85–95% for all seed lots (data not shown). Therefore, to compare the response of different seed batches to GS, half maximal effective concentrations (EC50) were modeled for each seed lot and reported in **Table S4**. Among the four GR24 stereoisomers, (+)-GR24 and (-)-2’-*epi*-GR24, which present natural stereochemical forms, showed the lowest EC50 and were the most active on all parasitic seeds (**Figure 2**). The other enantiomeres, (-)-GR24, which is usually associated to KAI2-Ligand activity, and (+)-2’-*epi*-GR24 were 1000 times less active than (+)-GR24 and (-)-2’-*epi*-GR24 except on seeds that were harvested in tobacco fields. Parasite seed lots from tobacco plots showed a median sensitivity to (+)-GR24: 100 times less active than (-)-2’-*epi*-GR24, but 10 times more active than (-)-GR24 and (+)-2’-*epi*-GR24. As the racemic mix was composed of (+)-GR24 and (-)-GR24, activities of (±)-GR24 highlights that of the highest active compound, (+)-GR24 enantiomer.

Seeds originating from hemp plots, genetic group 2a, were 10 to 100-fold less sensitive to the four GR24 stereoisomers than seeds originating from oilseed rape or tobacco plots (**Figure 2**). However, no difference was observed between the seeds originating from oilseed rape plots, of genetic group 1, and tobacco plots except for the (+)-GR24 (100 fold less active for tobacco).

The analysis of seed GS sensitivity in relation to the genetic distance of seed lots highlighted homogeneous responses within genetic groups (**Figures S2** and **S3**). For instance, seeds of genetic group 1 showed a high sensitivity towards (+)-GR24 while seeds of genetic group 2a showed a low sensitivity. Those data indicated that parasite seeds originating from hemp, of genetic group 2a, lost sensitivity towards (-)-GR24, (+)-2’-*epi*-GR24 and (-)-2’-*epi*-GR24 in regards to other samples.

Regarding responses to 2-PEITC, parasite seeds from hemp plots, of genetic group 2a, were less sensitive compared to seeds from oilseed rape plots, of genetic group 1, while seeds from tobacco plots showed an intermediate sensitivity: respectively 2.08 x10^-7^M (± 1.72 x10^-7^), 2.38 x10^-8^M (± 1.21 x10^-8^) and 1.43 x10^-7^M (± 1.78 x10^-7^). However, responses of seeds originating from tobacco and hemp plots, of genetic group 2a, were very heterogenous. On the other hand, all seed lots originating from oilseed rape plots, of genetic group 1, were homogeneously sensitive to low concentrations of 2-PEITC (**Figure S3**).

### Sequencing Features

The sequencing output contained 20,431,764 reads of which 79.4% of the sequenced nucleotides had a quality greater than or equal to Q30. Before proceeding to the diversity analysis, contaminant ASV, control samples and chloroplast ASV were removed from the raw dataset. Within the cleaned dataset, the number of reads per sample ranged from 7,170 to 16,384 reads for the 16S dataset and from 1,222 to 29,509 for the ITS dataset. The sample size median was at 10,352 with a standard deviation of 1,749.66 for 16S and 7,958 ± 2,677.55 for ITS.

### Microbial Alpha Diversity of Seeds Was Relatively Homogeneous

**Figure 3:**
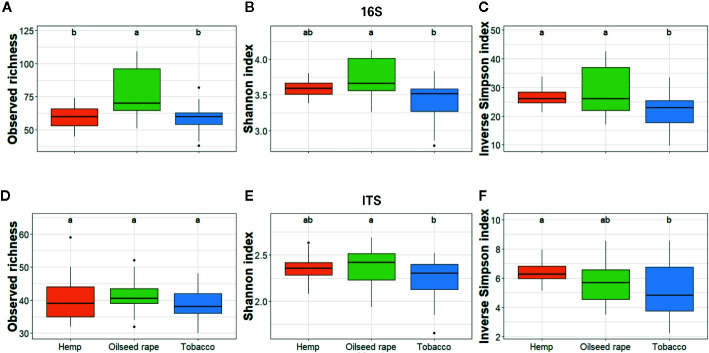
Alpha diversity of bacterial and fungal seed communities. It was estimated as the Observed richness **(A, D)**, Simpson index **(B, E)**, and Inverse Shannon index **(C, F)** for both 16S **(A–C)** and ITS **(D–F)**. The alpha diversity was measured for each biological replicate and is displayed per host of sampling. Different lowercase letters represent statistically different groups (Anova with post-hoc Tukey, p-value < 0.05).

Among seed samples, bacterial alpha diversity which represents the mean ASV diversity per sample, expressed by its richness (number of ASV in a sample) and its evenness (how close ASV numbers are close one to another in a sample), was homogeneous except for five samples out of 26 samples. Three samples from the first plot where oilseed rape was cultivated had a higher richness within the three indices than other samples. Two samples from two different tobacco plots had a lower richness with the three indices than other samples (**Figure S4**). Therefore, when assessing alpha diversity by host, statistical differences for the three indices used were observed only between oilseed rape and tobacco hosts within the bacterial community: seeds from oilseed rape plots had a higher bacterial alpha diversity than seeds from tobacco plots (**Figure 3**). Besides, when assessing alpha diversity for the fungal microbiota, no statistical differences were observed between originating host.

### Parasitic Seed Microbial Communities Clustered According to Their Originating Host

**Figure 4:**
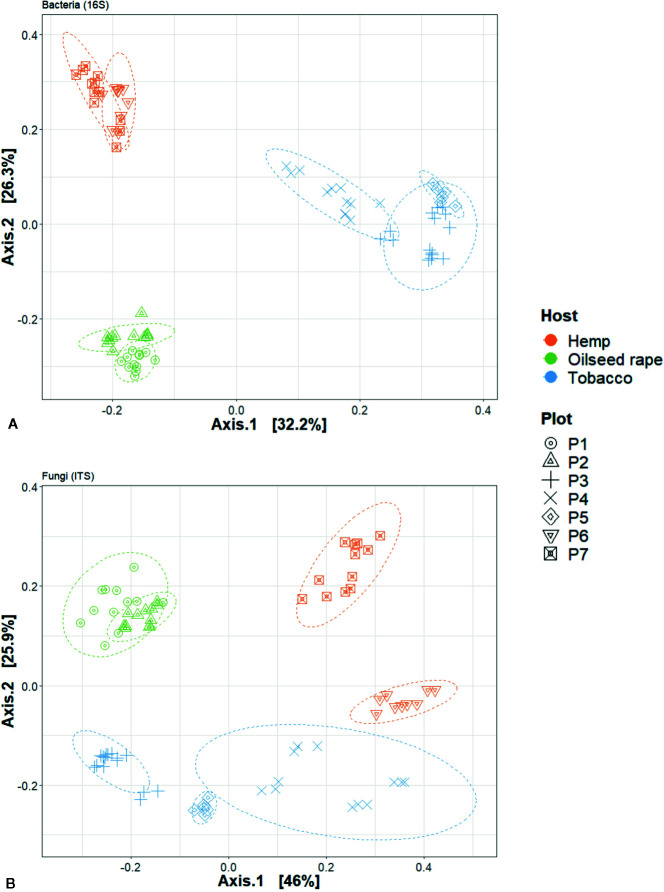
Principal Coordinate Analysis (PCoA) analysis of seed microbial communities based on Bray-curtis index regarding hosts and plots of origin. This is dislpayed for **(A)** 16S and **(B)** internal transcribed spacer (ITS) data. Each point represents one biological replicate in the PCoA scatterplot.

We used a principal coordinate analysis based on a Bray-Curtis distance matrix to determine the dissimilarity of microbial communities between samples (**Figure 4**). Overall, seed microbial communities were clustered according to their originating host and plot. For 16S, the first and second dimensions explained 32.2% and 26.3% of the data dispersion respectively. Seed samples from tobacco plots were separated from other seed samples within the first dimension and seed samples originating from oilseed rape and hemp plots were separated from each other along the second dimension. Within host clusters, there were also distinct but close sub-clusters for each plot. For ITS, the first and second dimensions explained 46% and 25.9% of the data dispersion respectively. Seed samples were strongly clustered both by host and by plot of origin. Looking at the clusters, the seed samples originating from oilseed rape plots showed the most homogeneous microbial communities while the seed samples originating from tobacco plots were the most heterogeneous. Seed samples P4S1, P4S2, P4S3, and P4S4 which had a genetic distance to the Pram120 reference between 0.47 and 0.49 clustered together. On the other hand, seed samples P3S1, P3S2, P3S4, P3S5 together with samples P5S1 and P5S2 which had a higher genetic distance to Pram120 between 0.51 - 0.53 were also sub-clustered (**Figure S5**). The similarity of the microbial profiles of plots P3 and P5 did not reflect the geographical proximity between these plots, because P3 was geographically distant from the mirroring plots P4 and P5 (about 30 km away). Both bacterial and fungal Bray-Curtis distances were highly explained by either independent or combined host, plot, and MLG (*P. ramosa* multilocus genotypes) qualitative variables as all tested models were significant (**Table 1**). As those variables were nested, there is an increasing effect of the variables on the dispersion (Host R^2^< Plot R^2^< MLG R^2^) as displayed by plot and MLG subclusters in the PCoA (**Figure 4**, **Figure S5**). Also interactive effects between host, plot and genotype explained most of the data variability with 86% and 94% of the variability respectively for bacteria and fungi.

**Table 1:** Permutation test on bacterial and fungal Bray-Curtis distance-based redundancy analysis based on host, plots, and multilocus genotype (MLG) groups as constraints.

		df^a^	SS^b^	F	p-value	MS^c^	Adjusted R²
**Bacteria - 16S**							
** Host x Plot x MLG**	Model	17	11.306	23.04	<0.001	0.66506	0.86
	Residual	60	1.732			0.02887	
** Host**	Model	2	7.0303	43.88	<0.001	3.51515	0.54
	Residual	75	6.0081			0.08011	
** Plot**	Model	6	9.8665	36.809	<0.001	1.64442	0.76
	Residual	71	3.1719			0.04467	
** MLG**	Model	16	10.796	18.36	<0.001	0.67475	0.81
	Residual	61	2.242			0.03675	
**Fungi - ITS1**							
** Host x Plot x MLG**	Model	17	9.2153	34.8	<0.001	0.54208	0.94
	Residual	60	0.9346			0.01558	
** Host**	Model	2	5.2187	39.686	<0.001	2.60935	0.53
	Residual	75	4.9313			0.06575	
** Plot**	Model	6	8.2764	52.273	<0.001	1.3794	0.84
	Residual	71	1.8736			0.02639	
** MLG**	Model	16	8.9782	29.211	<0.001	0.56114	0.91
	Residual	61	1.1718			0.01921	

a Degree of freedom.

b Sum of squares.

c Mean of squares.

### *P. ramosa* Seed Core Microbiota

Regarding 16S assignation, 98.6% of the ASVs belonged to the Bacteria domain. Four ASVs were part of the Archaea domain and 5 ASVs were unassigned. Within these domains, four phyla that represented 99.76% of the total abundance and 90.5% of ASVs, were present in 100% of the samples. In order of abundance, they were: *Proteobacteria* (64.54% of the total abundance and 40.5% of ASVs), *Bacteroidetes* (29.64% and 35.82%), *Actinobacteria* (3.37% and 9.97%) and *Firmicutes* (2.21% and 4.21%). At the genus level, seven genera representing 63.47% of the total abundance and gathered 23.05% of the ASVs and were present in 100% of the samples triplicate: *Pseudomonas* (19.85% of the total abundance and 3.27% of the ASVs), *Pedobacter* (10.45% and 4.98%), *Stenotrophomonas* (10.11% and 1.25%), *Chryseobacterium* (9.82% and 2.65%), *Sphingobacterium* (6.45% and 6.39%), *Rhizobium* (3.58% and 1.71%) and *Sphingomonas* (3.21% and 2.80%). Additionally, 14.3% of the ASVs were unidentified representing 19.84% of the total abundance. However, these ASVs were numerous and diverse but at low abundance because their median abundance was zero among all samples.

Regarding ITS assignation, all ASVs belonged to the fungi kingdom. At the phylum level, 13.09% of the ASV were unidentified, representing 1.74% of the total abundance. Unidentified fungi phyla had a median abundance of 60.5. Besides these unidentified phyla, two phyla representing 98.24% of the total abundance with 84.68% of the ASVs and were present in every sample: *Ascomycota* (91.75% of the total abundance and 64.62% of ASVs) and *Basidiomycota* (6.50% and 20.06%). At the genus level, 40.11% of the ASVs were unidentified, representing 22.73% of the total abundance (median abundance at zero). Moreover, six genera were present in every sample triplicate representing 64.43% of the total abundance and 11.98% of the ASVs: *Cladosporium* (33.19% of the total abundance and 1.11% of the ASVs), *Fusarium* (6.81% and 4.46%), *Gliocladium* (4.49% and 1.95%), *Mycosphaerella* (2.67% and 0.28%), *Plectosphaerella* (13.48% and 1.39%), and *Vishniacozyma* (3.78% and 2.78%).

These seven bacterial genera and six fungi genera were thus part of the seed core microbiota. To identify more precisely the seed core microbiota, we looked at the ASVs present in every seed sample. Regarding the bacterial community, ten ASVs were present in every samples representing 1.56% of the ASVs and 26.46% of the total abundance (**Figure S6**). Regarding the fungal community, seven ASVs were present in every sample representing 1.95% of the ASVs and 60.91% of the total abundance (**Figure S7**). Within these 17 ASVs (**Table 2**), three were unidentified, nine were identified at the genus level and five were identified at the species level, by reference to the Silva or UNITE taxonomic databases for the 16S ASVs and the ITS ASVs respectively. The ASV sequences were compared to the NCBI database (BLAST). The best hits were listed in **Table 2**. Note that ASV2639 identified as *Mycosphaerella tassiana* in UNITE database was identified as *Cladosporium floccosum* in the NCBI database.

**Table 2:** All *P. ramosa* seed core microbial Amplicon Sequence Variants (ASVs) taxonomic assignation and abundance.

Marker Gene	ASV identifier and taxonomic identification on SILVA^a^ and UNITE^b^ databases	Relative abundance^c^ (% of the total abundance)	Top hit BLAST onto NCBI database
**16S**	ASV2990 *Pseudomonas* unidentified	5.55	*Pseudomonas viridiflava* *Pseudomonas rhizosphaerae* *Pseudomonas abietaniphila* *Pseudomonas graminis* *Pseudomonas lutea* *Pseudomonas donghuensis*
	ASV2265 *Stenotrophomonas* unidentified	4.09	*Stenotrophomonas tumulicola* *Stenotrophomonas maltophilia*
	ASV226 *Stenotrophomonas* *rhizophila*	4.07	
	ASV2956 *Pseudomonas* unidentified	2.45	*Pseudomonas fluorescens* *Pseudomonas koreensis* *Pseudomonas baetica*
	ASV1971 *Rhizobium* unidentified	2.41	*Rhizobium radiobacter* *Agrobacterium tumefaciens*
	ASV710 *Pedobacter* unidentified	2.31	*Pedobacter roseus* *Pedobacter suwonensis* *Pedobacter agri* *Pedobacter borealis* *Pedobacter humicola* *Pedobacter terrae*
	ASV1578 *Sphingomonas* unidentified	1.92	*Sphingomonas faeni* *Sphingomonas olei*
	ASV2268 *Stenotrophomonas* *chelatiphaga*	1.52	
	ASV2876 *Paenibacillus* unidentified	1.48	*Paenibacillus illinoisensis* *Paenibacillus hordei* *Paenibacillus kyungheensis*
	ASV1998 unidentified	0.66	*Rhodobacter* sp.
**ITS**	ASV2644 *Cladosporium* unidentified	32.82	*Cladosporium cladosporioides* *Cladosporium tenuissimum*
	ASV1920 *Plectosphaerella* *cucumerina*	13.34	
	ASV2459 unidentified	5.18	*Fusarium avenaceum* *Fusarium acuminatum*
	ASV1817 unidentified	2.72	*Alternaria infectoria*
	ASV2639 *Mycosphaerella tassiana*	2.67	*Cladosporium floccosum*
	ASV2467 *Fusarium* unidentified	2.33	*Fusarium oxysporum*
	ASV140 *Vishniacozyma* *victoriae*	1.85	

a SILVA, database of bacterial species (https://www.arb-silva.de/).

b UNITE, database of fungal species (https://unite.ut.ee/).

c Relative abundance represents the abundance sum of each seed core ASV expressed as percentage to the total abundance in all samples.

### Some Seed ASVs Were Specific to Their Parasitized Host

As some ASVs were present in every samples, most ASVs were only found in one or some samples. In fact, each sample mostly revealed unique ASVs ranging from 7 to 27 and 2 to 20 per sample of bacteria and fungi respectively. In average, about 13.15 ASVs for bacteria and 6.95 ASVs for fungi were unique per samples with low abundances (**Figures S6** and **S7**). Considering the host specialization of *P. ramosa*, we looked at the ASV only present in one of the originating hosts (**Table 3**). Two bacterial ASVs and two fungal ASVs were found in every samples originating from oilseed rape plots and not in the other samples: bacterial ASV668 (*Sphingobacterium* sp. MIMdw12), bacterial ASV3317 (*Nannocystis* sp.), fungal ASV1153 (*Gymnochlora stellate*) and fungal ASV1708 (*Leptosphaeria maculans*). One bacterial ASV was found in every samples originating from hemp plots and not in the other samples: bacterial ASV718 (*Pedobacter* sp.). Two bacterial ASVs were found in every samples originating from tobacco plots and not in the other samples: bacterial ASV638 (*Sphingobacterium* sp. 23D10-4-9) and bacterial ASV2965 (*Pseudomonas* sp.).

**Table 3:** Host related – *P. ramosa* seed core microbial Amplicon Sequence Variants (ASVs) taxonomic assignation and abundance.

MarkerGene	Originating host	ASV identifier and taxonomic identification on SILVA^a^ and UNITE^b^ databases	Relative abundance^c^ (% of the total abundance)	Top hit BLAST
**16S**	Oilseed rape	ASV668 *Sphingobacterium* sp. MIMdw12	0.26	*Sphingobacterium spiritivorum*
		ASV3317 *Nannocystis* unidentified	0.47	*Nannocystis pusilla* *Nannocystis exedens*
	Hemp	ASV718 *Pedobacter* unidentified	0.27	*Pedobacter jejuensis* *Pedobacter kribbensis*
	Tobacco	ASV638 *Sphingobacterium* sp. 23D10-4-9	0.33	*Sphingobacterium corticis* *Sphingobacterium populi*
		ASV2965 *Pseudomonas* unidentified	2.41	*Pseudomonas putida* *Pseudomonas plecoglossicida*
**ITS**	Oilseed rape	ASV1153 unidentified	0.04	*Gymnochlora stellate*
		ASV1708 *Leptosphaeria maculans*	0.03	*Leptosphaeria maculans*

a SILVA, database of bacterial species (https://www.arb-silva.de/).

b UNITE, database of fungal species (https://unite.ut.ee/).

^c^Relative abundance represents the abundance sum of each seed core ASV expressed as percentage to the total abundance in all samples.

### Seed Core ASV Were Also Found in Soil

Seed microbiota and soil microbiota compositions were relatively different, as they clustered strongly for bacteria and slightly less for fungi on the first axis of the PCoA based on Bray-Curtis indices with 37.4% and 26.8% of data dispersion explained respectively (**Figure S8**).

Looking at the seed core microbiota, all ASVs were also found in soil samples. Bacterial seed-core ASVs were present in only 6.06% to 45.45% of all sampled soils in low relative abundance ranging from 0.01% to 0.52% of the total soil ASV abundance (**Table S5**, **Figure S9**). Fungal seed-core ASVs were over represented in soils as their prevalence ranged from 89.9 to 100% of all samples but also showed limited abundance compared to others ASVs ranging from 0.84% to 8.95% of all soil ASVs (**Table S5**, **Figure S9**). However, looking a the raw abundances, core bacterial ASV were about 4.67 to 97.88 times more numerous in seed than soils while core fungal ASV were similar as the ratio seed - soil ranged from 0.34 to 7.00 (**Table S5**).

Regarding the host related – *P. ramosa* seed core microbial ASVs, six out of seven ASV were found in soil samples (**Table S6**). The relative abundance and the relative prevalence of every host related – *P. ramosa* seed core bacterial ASV were higher in seed samples than in soil samples. Indeed, seed core bacterial ASV prevalence ranged from 0% to 6.06% in soil samples corresponding to one crop type and their abundance was lower than 0.01% of the total ASV abundance (**Table S6**, **Figure S9**). On the other hand, the relative abundances of seed core fungal ASVs related to hosts were smaller in seed samples than in soil samples while their relative prevalences were higher in seed samples than in soil samples. The two fungal seed-core ASVs that were retrieved in oilseed rape plots were relatively well found in oilseed rape soils with prevalences of 27.27% and 16.67% but low relative abundances of 0.14% and 0.58% of the total ASVs, respectively for ASV1153 (*Gymnochlora stellate*) and ASV1708 (*Leptosphaeria maculans*) (**Table S6**). However, looking at raw abundances, ASV1153 and ASV1708 showed a seed/soil ratio of 0.07 and 0.39, which suggested 10–100 times higher raw abundances in soils than in seeds (**Table S6**).

## Discussion

### Seed Characterization and Host Specialization

Seeds of *P. ramosa* collected in this study were harvested on three parasitized hosts and two monophyletic groups were differentiated for oilseed rape and hemp, respectively, corresponding to groups 1 and 2a while parasite seeds from tobacco plots were paraphyletic (Stojanova et al., 2019). As rhizospheres of the studied host plants harbor different metabolites in different quantities, they possibly induce differently the germination of parasitic seeds. Indeed oilseed rape and *Brassicaceae* rhizosphere is characterized by the presence of glucosinolates and ITC (Ishida et al., 2014; Xu et al., 2017)⁠ but no strigolactone has been yet confirmed (Auger et al., 2012; Yoneyama et al., 2018)⁠. This absence or undetectable presence of strigolactones is to be related to the absence of mycorrhizal association of *Brassicacaeae* with fungi as strigolactones are a necessary signal to initiate the symbiosis. On the other hand, tobacco plants exude different types of strigolactones associated to (+)-GR24 and (-)-2’-*epi*-GR24 while no known canonic strigolactones has been detected in hemp exudates (personal communication).

Seed sensitivity to different GS is a good proxy of host specialization. Indeed, we showed that parasitic seeds of genetic group 1 were highly sensitive to strigolactone synthetic enantiomers as they were able to germinate with up to 10^-12^M of (+)-GR24 and (-)-2’-*epi*-GR24. This was consistent with previous work that showed that *P. ramosa* seeds from oilseed rape plots were ten to hundred times more sensitive to (±)-GR24 and to different conformations of GR24 than seeds from hemp plots (Boyer et al., 2014; Dvorakova et al., 2018; Dvorakova et al., 2019)⁠. Also, seeds from oilseed rape and hemp plots perceived at a lower concentration the (+)-GR24 and (-)-2’-*epi*-GR24, and were less sensitive to GR24 with non-natural conformation (de Saint Germain et al., 2019)⁠. Seeds originating from tobacco plots, which had a greater genetic diversity, showed also more diverse and intermediate responses to GS, which corroborates its generalist parasitic status. However, all seed lots originating from tobacco plots were a hundred times more sensitive to (-)-2’-*epi*-GR24 than to (+)-GR24, contrary to seeds from oilseed rape and hemp plots which showed similar sensitivities to these two forms. This could show a different adaptation of those seeds to the tobacco cultivars which harbor both 4-deoxyorobanchol and 5-deoxystrigol in their root exudates, respectively corresponding to the synthetic isoforms 2’-*epi*-GR24 and (+)-GR24 (Xie et al., 2013; Xie, 2016)⁠.

On the other hand, while the response to 2-PEITC was similar for two *P. ramosa* seed lots harvested in oilseed rape and hemp fields in a previous work (de Saint Germain et al., 2019)⁠, the present study included more seed diversity and showed a significant different response for *P. ramosa* seeds from oilseed rape and hemp plots. Highly sensitive physiological responses of oilseed rape plot -originating seeds, of genetic group 1, may have emerged due to the presence of ITC in oilseed rape rhizosphere combined with reduced quantities of strigolactones (Auger et al., 2012)⁠. As 2-PEITC and GR24 molecules induce the same physiological responses in *P. ramosa* seeds of genetic group 1 (Brun et al., 2019)⁠, the receptors for both types of molecules could be related paralogs with different affinities. Also, the highly heterogeneous sensitivity of seeds originating from tobacco and hemp plots, of genetic group 2a, in response to 2-PEITC suggested a specialization within those genetic groups. Finally, as seeds of group 2a showed weaker sensitivities to any tested GS, they could be adapted to other yet unknown hemp produced GS.

Evolutionary speaking, these data suggest an adaptation of GS receptors in connection with host specialization between and within *P. ramosa* populations. Hence the parasitic plant diversity and host specialization may have been driven by rhizosphere GS profiles.

### Factors Influencing Microbial Composition of Parasitic Seeds

In the present study, we also showed that microbial communities from parasitic seeds were influenced by the host, the plot and the parasitic plant genotype. Also, the plot effect was more important for fungal than for bacterial communities. Similar observations were made several times on crop seeds and indicated that seed-fungal communities were rather transmitted horizontally by the environment and soil versus seed-bacterial communities which had mostly a vertical transmission (Klaedtke et al., 2016)⁠. Here the environment horizontal transmission is most likely due to environmental microbial deposition on flowers and fruits and less likely due to pollinators as *P. ramosa* is mostly autogamous (Parker and Riches, 1993; Vaz Patto et al., 2009). In addition, the parasitic vascular connection to the host certainly plays an important role in the final seed community composition in the parasitic plant. As previously evidenced for the close species, *Phelipanche aegyptiaca* and *Orobanche hederae*, there is a flow of microorganisms and a homogenization between the host and the parasite during the interaction (Iasur Kruh et al., 2017; Fitzpatrick and Schneider, 2020). Finally, in the present study, we overlooked the direct soil contamination, which happens when seeds are disseminated in the soil where they can lie for decades. Soil microbiota compete with seed-born pioneer endophytes, which may undergo other selection processes that alter the microbial profiles.

Also, there were indications that genetic diversity of seeds had an effect on the assemblage of seed microbial communities. For both bacteria and fungi, tobacco and hemp plot -originating seeds which comprised genetic diversity, communities were sub-clustered regarding their genetic distance to *Pram120* which tends to indicate also a genetic effect confirmed by adjusted R² calculations. Indeed, even though MLG variable is superposed with host and plot variables, there is more variability explained by the MLGs than by the other single variables. Intra-species genotypes are known to play a substantial role in the selection and structure of microbial communities in the plant rhizosphere and endosphere but also on the seed endophytic community (Sapkota et al., 2015; Gallart et al., 2018; Walitang et al., 2018). As no genetic group was sampled from different hosts, the genetic group effect and the host effect were not differentiated on the microbial structure. Extensive and massive population genetic approaches coupled with experimental evolution approaches would better describe host vs genetic group effect in each plot. Either (i) there are parasitic seeds of different genetic groups in the soil and the above ground genetic groups arise from the present crop cultivation preference or (ii) there is only one seed genetic group in the soil due to long-term adaptation to the intensive local crop cultivation. In fact, no cross cultivation of any other host type were recorded on the studied plot for at least ten years preceding sampling (**Table S2**).

### Ubiquitous Core ASVs in Parasitic Seeds

Overall mostly *Proteobacteria*, *Actinobacteria*, *Firmicutes*, and *Bacteroidetes* bacterial phyla and *Basidiomycetes* and *Ascomycetes* fungal phyla were found in seeds. Their presence and abundance are in accordance with usual seed phyla due to their great importance in the environment. They are especially dominant in soil, which leads to a high probability for any seed to encounter such taxa (Shade et al., 2017). Regarding core seed microbiota, fungal ASVs which came forth as of *Fusarium*, *Alternaria, Cladosporium, Plectosphaerella*, and Vishniacozyma genera, were also found in the surrounding soil and are known to be predominantly present in soils and can be maintained on bare soils after crop harvest or upon crop rotation (Palm and Rotem, 1997; Smith, 2007; Bensch et al., 2012; Mašínová et al., 2017; Giraldo and Crous, 2019)⁠. *Alternaria* and *Fusarium* have been largely reported in crop and natural seeds in great abundance (Verma and White, 2019)⁠. Additionally, beside the basidiomycetous yeast Vishniacozyma, all genus are also described as opportunist pathogenic taxa that are able to colonize plant tissues and seeds asymptomatically (Thomma, 2003; Perelló and Larrán, 2013; Links et al., 2014; Raimondo and Carlucci, 2018; Henry et al., 2019). Possibly the core ASVs also have beneficial properties for plants and in particular for *P. ramosa* and it is unclear whether these taxa persist as ubiquitous endophytes due to the fungal dispersal strategy or as a plant strategy to supply beneficial microbes for a better offspring fitness (Verma and White, 2019)⁠. In fact, there are a few evidences of plant-beneficial capacity of *Fusarium, Altenaria*, and *Cladosporium* species against pathogen and pests (Atkins et al., 2003; Hamayun et al., 2010; Lorincz et al., 2010; Murphy and Doohan, 2018; Noor et al., 2018; Pappas et al., 2018). As examples, biocontrol activities of endophytic *Fusarium* spp., the most studied genus, have been reported against bacterial plant pathogens including *Pseudomonas aeruginosa* (Schulz et al., 2015), *Microbotryum violaceum* or even *against* other phytopathogenic *Fusarium via* antimicrobial compounds (Hussain et al., 2015) and/or by niche competition (Marín et al., 1998)⁠. As *Fusarium* produce a number of phytotoxic molecules (Nelson et al., 1993; Nelson et al., 1994)⁠, *Fusarium* metabolites have also been considered for *P. ramosa* biocontrol with low expectations of massive and efficient transfer to agricultural systems (Cohen et al., 2002; Hasannejad et al., 2006)⁠⁠.

On the other hand, for bacteria, only three core ASVs also were recovered in the surrounding soil while seven ASVs were then only present in seeds. The three ASVs retrieved in soil were related to, in order of abundance, *Pseudomonas*, *Stenotrophomonas*, and *Pedobacter* genera which have previously been described in plants and soils (Steyn et al., 1998; Sørensen and Nybroe, 2004; Ryan et al., 2009)⁠. The other ASVs were related to, in order of abundance, *Stenotrophomonas*, *Pseudomonas*, *Rhizobium*, *Sphingomonas*, *Paenibacillus*, and *Rhodobacter* genera. All of them can usually be found as endophytes in plants but also in the near environment like the rhizosphere and bulk soils (Cocking, 2003; Rosenblueth and Martínez-Romero, 2006; Ryan et al., 2009; Johnston-Monje and Raizada, 2011; Oteino et al., 2015; Maida et al., 2016)⁠. Core bacterial ASVs are most likely endophytic strains that were either transmitted within the parasitic plant but also from the host plant, or diffused and assimilated from the soil. At the seed level, *Paenibacillus*, *Pseudomonas*, *Rhizobium*, and *Sphingomonas* have been repeatedly described as abundant endophytes in crop seeds while *Stenotrophomonas* was described as a less abundant but common seed endophytic genus (Verma and White, 2019)⁠. Particularly, *Pseudomonas* species have very often been described in crop seeds of both dicotyledons and monocotyledons (Johnston-Monje and Raizada, 2011; Hardoim et al., 2012)⁠. In oilseed rape seeds especially, strains of *Pseudomonas* are omnipresent (Barret et al., 2015; Rezki et al., 2016). Additionally, it is widely reported that *Pseudomonas* species and particularly *P. fluorescent* have plant growth promoting activities (Patten and Glick, 2002; Dey et al., 2004; Großkinsky et al., 2016)⁠. Furthermore, Plant Growth-Promoting Rhizobacteria (PGPR) - nitrogen fixing ability has been described for *Pseudomonas*, *Rhizobium* and *Rhodobacter* species and phosphate solubilization ability for *Paenibacillus*, *Pseudomonas*, and *Rhizobium* species (Hayat et al., 2012).

### Host-Related Core ASVs Likely Involved in the Parasitic Interaction

ASVs that were shared by all *P. ramosa* seed samples among host groups ranged from one in hemp plot -originating seeds to four in oilseed rape plot –originating seeds. All host seed groups had at least one core bacterial ASV while only oilseed rape plot -originating seeds showed a core fungal community. Whether those particular strains were recruited by the parasitic plants during their cycle and involved in the seed physiology or whether they co-evolved with their associated genotypic group and host cannot be determined. Also, it has to be highlighted that those host related core ASVs are present in the west of France and may be absent in any other region. Parasite seeds from hemp plots harbored one bacterial core ASV in high abundance that belonged to *Pedobacter* genus. This was consistent with a couple or reports that spotted *Pedobacter* species in hemp fibers during retting process (Fu et al., 2011; Ribeiro et al., 2015; Liu et al., 2017). However, no so much is known for the related to top hit species *Pedobacter* *jejuensis* or *Pedobacter* *kribbensis*. Additionally, parasite seeds from tobacco plots harbored two abundant core bacterial ASVs, related to *Sphingobacterium* (spp. *corticis* or *populi*) and *Pseudomonas* (spp. *putida* or *plecoglossicida*) which are ubiquitous seed endophytic genera. In the past years, novel *Sphingobacterium* species have been isolated in tobacco rhizosphere and roots several times (Liu et al., 2012; Zhou et al., 2017). Moreover, *Sphingobacterium* sp., *Sphingobacterium* *multivorum*, *Pseudomonas* sp., and *Pseudomonas* putida strains have been described in tobacco plots as nicotine degraders which can durably live as endophytes (Ma et al., 2012)⁠. From oilseed rape plots, parasite seeds harbor two core bacterial ASVs, namely *Sphingobacterium* *spiritivorum* and *Nannocystis* sp. (*pusilla* or *exedens*), and two fungal ASVs, namely *Leptosphaeria* *maculans* and *Gymnochlora* *stellate*. The *Nannocystis* sp. and *Gymnochlora* *stellate* related ASVs were in abundance but those taxa are rarely found in nature – and thus understudied – which might indicate some sort of high specialization for oilseed rape related – *P. ramosa* seeds. On the other hand, *Sphingobacterium* species and *Leptosphaeria maculans* are quite commonly described and found. Interestingly, one *Sphingobacterium* strain showed a myrosinase activity (Meulenbeld and Hartmans, 2001)⁠, which suggests a mutualistic interaction between the bacterial core *Sphingobacterium* species and *P. ramosa* seeds helping to produce germination stimulants through glucosinolate breakdown and then ITC release in the oilseed rape rhizosphere. Also, *Leptosphaeria maculans*, a phytopathogenic agent that causes diseases on *Brassica* species, is known to trigger an increase in glucosinolate production as a defense response in its *Brassica* hosts (Abdel-Farid et al., 2010; Robin et al., 2017). *L. maculans* related canker diseases have been reported since early 20th century in Europe, and then possibly cohabit with the parasitic weed *P. ramosa* in oilseed rape fields as they overlap in western France (Balesdent et al., 2006)⁠. Consequently, *P. ramosa* seeds of genetic group 1, which is an oilseed rape specialist, may have undergone a symbiotic co-evolution with this other oilseed rape pathogen. Ultimately, one can speculate that *P. ramosa* endophytes *L. maculans*, triggering glucosinolate induction in oilseed rape, and *Sphingobacterium* sp., metabolizing glucosinolate metabolites into ITC, could be co-selected for an improved parasitic seed fitness and recognition of its host. This proposed adapted–seed–microbiota model should be further analyzed to be biologically corroborated.

In conclusion, this work pictured the microbial communities that are carried and selected by *P. ramosa* seeds with regard to their host. Host type was shown to structure microorganisms whether they are selected and transmitted from the soil or from the plants – host or parasite. *P. ramosa* seeds harbored specific core microorganisms that may be essential for the dialogue with the host plant. Especially seed of the genetic group 1, showing adaptation to oilseed rape, seemed to harbor a rather adapted microbiota to the parasitic plant – host plant interaction.

## Data Availability Statement

The datasets generated for this study can be found in the https://www.ncbi.nlm.nih.gov/bioproject/PRJNA589539.

## Author Contributions

LP, J-BP, PD, and PS designed the project and acquired the funds. This work results from the master thesis of SH who was supervised by LP and J-BP. SH, SD, J-BP, and LP performed the lab experiments. SH, CM, and MB performed the library preparation and Mi-Seq sequencing. ED provided bioinformatics facilities and help through the BiRD platform of Biogenouest. SH, J-BP, and LP analyzed the results. SH, J-BP, and LP wrote the article. PS and PD corrected and reviewed the article. All authors contributed to the article and approved the submitted version.

## Funding

This article received support from a RFI Objectif Végétal Starter grant allocated by the Pays de la Loire Regional Council. This research was conducted in the framework of the regional programme “Objectif Végétal, Research, Education and Innovation in Pays de la Loire”, supported by the French Region Pays de la Loire, Angers Loire Métropole and the European Regional Development Fund.

## Conflict of Interest

The authors declare that the research was conducted in the absence of any commercial or financial relationships that could be construed as a potential conflict of interest.

## Footnotes

1 Available at: https://www.bioinformatics.babraham.ac.uk/projects/fastqc.

## References

[B1] Abdel-Farid I. B. Jahangir M. Mustafa N. R. van Dam N. M. van den Hondel C. A. M. J. J. Kim H. K. (2010). Glucosinolate profiling of Brassica rapa cultivars after infection by Leptosphaeria maculans and Fusarium oxysporum. Biochem. Syst. Ecol. 38, 612–620. 10.1016/j.bse.2010.07.008

[B2] Albaser A. Kazana E. Bennett M. H. Cebeci F. Luang-In V. Spanu P. D. (2016). Discovery of a bacterial Glycoside Hydrolase family 3 (GH3) β-glucosidase with myrosinase activity from a citrobacter strain isolated from soil. J. Agric. Food Chem. 64, 1520–1527. 10.1021/acs.jafc.5b05381 26820976

[B3] Altschul S. F. Gish W. Miller W. Myers E. W. Lipman D. J. (1990). Basic local alignment search tool. J. Mol. Biol. 215, 403–410. 10.1016/S0022-2836(05)80360-2 2231712

[B4] Atkins S. D. Clark I. M. Sosnowska D. Hirsch P. R. Kerry B. R. (2003). Detection and quantification of Plectosphaerella cucumerina, a potential biological control agent of potato cyst nematodes, by using conventional PCR, real-time PCR, selective media, and baiting. Appl. Environ. Microbiol. 69, 4788–4793. 10.1128/AEM.69.8.4788-4793.2003 12902272 PMC169141

[B5] Auger B. Pouvreau J.-B. Pouponneau K. Yoneyama K. Montiel G. Le Bizec B. (2012). Germination stimulants of Phelipanche ramosa in the rhizosphere of Brassica napus are derived from the glucosinolate pathway. Mol. Plant-Microbe Interact. 25, 993–1004. 10.1094/MPMI-01-12-0006-R 22414435

[B6] Balesdent M. H. Louvard K. Pinochet X. Rouxel T. (2006). A large-scale survey of races of Leptosphaeria maculans occurring on oilseed rape in France. Eur. J. Plant Pathol. 114, 53–65. 10.1007/s10658-005-2104-0

[B7] Barret M. Briand M. Bonneau S. Préveaux A. Valière S. Bouchez O. (2015). Emergence shapes the structure of the seed microbiota. Appl. Environ. Microbiol. 81, 1257–1266. 10.1128/AEM.03722-14 25501471 PMC4309697

[B8] Beals E. W. (1984). Bray-Curtis ordination: an effective strategy for analysis of multivariate ecological data. Adv. Ecol. Res. 14, 1–55. 10.1016/S0065-2504(08)60168-3

[B9] Bensch K. Braun U. Groenewald J. Z. Crous P. W. (2012). The genus Cladosporium. Stud. Mycol. 72, 1–401. 10.3114/SIM0003 22815589 PMC3390897

[B10] Besserer A. Puech-Pagès V. Kiefer P. Gomez-Roldan V. Jauneau A. Roy S. (2006). Strigolactones stimulate arbuscular mycorrhizal fungi by activating mitochondria. PLoS Biol. 4, e226. 10.1371/journal.pbio.0040226 16787107 PMC1481526

[B11] Bolyen E. Rideout J. R. Dillon M. R. Bokulich N. A. Abnet C. C. Al-Ghalith G. A. (2019). Reproducible, interactive, scalable and extensible microbiome data science using QIIME 2. Nat. Biotechnol. 37, 852–857. 10.1038/s41587-019-0209-9 31341288 PMC7015180

[B12] Boyer F. D. De Saint Germain A. Pouvreau J. B. Clavé G. Pillot J. P. Roux A. (2014). New strigolactone analogs as plant hormones with low activities in the rhizosphere. Mol. Plant 7, 675–690. 10.1093/mp/sst163 24249726

[B13] Bray J. R. Curtis J. T. (1957). An ordination of the upland forest communities of Southern Wisconsin. Ecol. Monogr. 27, 325–349. 10.2307/1942268

[B14] Brun G. Thoiron S. Braem L. Pouvreau J.-B. Montiel G. Lechat M.-M. (2019). CYP707As are effectors of karrikin and strigolactone signalling pathways in Arabidopsis thaliana and parasitic plants. Plant Cell Environ. 42, 2612–2626. 10.1111/pce.13594 31134630

[B15] Bruvo R. Michiels N. K. D’Souza T. G. Schulenburg H. (2004). A simple method for the calculation of microsatellite genotype distances irrespective of ploidy level. Mol. Ecol. 13, 2101–2106. 10.1111/j.1365-294X.2004.02209.x 15189230

[B16] Buée M. Reich M. Murat C. Morin E. Nilsson R. H. Uroz S. (2009). 454 pyrosequencing analyses of forest soils reveal an unexpectedly high fungal diversity. New Phytol. 184, 449–456. 10.1111/j.1469-8137.2009.03003.x 19703112

[B17] Callahan B. J. McMurdie P. J. Rosen M. J. Han A. W. Johnson A. J. A. Holmes S. P. (2016). DADA2: High-resolution sample inference from Illumina amplicon data. Nat. Methods 13, 581–583. 10.1038/nmeth.3869 27214047 PMC4927377

[B18] Cankar K. Kraigher H. Ravnikar M. Rupnik M. (2005). Bacterial endophytes from seeds of Norway spruce (Picea abies L. Karst). FEMS Microbiol. Lett. 244, 341–345. 10.1016/j.femsle.2005.02.008 15766788

[B19] Caporaso J. G. Lauber C. L. Walters W. A. Berg-Lyons D. Lozupone C. A. Turnbaugh P. J. (2011). Global patterns of 16S rRNA diversity at a depth of millions of sequences per sample. Proc. Natl. Acad. Sci. U. S. A. 108 (Suppl), 4516–4522. 10.1073/pnas.1000080107 20534432 PMC3063599

[B20] Castejon M. Romero-Munoz F. Garcia-Torresl L. (1991). Orobanche cernua seed dispersal through sunflower achenes. Helia 14, 51–54.

[B21] Cocking E. C. (2003). Endophytic colonization of plant roots by nitrogen-fixing bacteria. Plant Soil 252, 169–175. 10.1023/A:1024106605806

[B22] Cohen B. A. Amsellem Z. Lev-Yadun S. Gressel J. (2002). Infection of tubercles of the parasitic weed Orobanche aegyptiaca by mycoherbicidal Fusarium species. Ann. Bot. 90, 567–578. 10.1093/aob/mcf238 12466097 PMC4240454

[B23] Conn C. E. Bythell-Douglas R. Neumann D. Yoshida S. Whittington B. Westwood J. H. (2015). Convergent evolution of strigolactone perception enabled host detection in parasitic plants. Science 349, 540–543. 10.1126/science.aab1140 26228149

[B24] Covarrubias-Pazaran G. Diaz-Garcia L. Schlautman B. Salazar W. Zalapa J. (2016). Fragman: an R package for fragment analysis. BMC Genet. 17, 62. 10.1186/s12863-016-0365-6 27098093 PMC4839125

[B25] Davis N. M. Proctor D. M. Holmes S. P. Relman D. A. Callahan B. J. (2018). Simple statistical identification and removal of contaminant sequences in marker-gene and metagenomics data. Microbiome 6, 226. 10.1186/s40168-018-0605-2 30558668 PMC6298009

[B26] de Saint Germain A. Retailleau P. Norsikian S. Servajean V. Pelissier F. Steinmetz V. (2019). Contalactone, a contaminant formed during chemical synthesis of the strigolactone reference GR24 is also a strigolactone mimic. Phytochemistry 168, 112112. 10.1016/j.phytochem.2019.112112 31499274

[B27] Delaux P.-M. Xie X. Timme R. E. Puech-Pages V. Dunand C. Lecompte E. (2012). Origin of strigolactones in the green lineage. New Phytol. 195, 857–871. 10.1111/j.1469-8137.2012.04209.x 22738134

[B28] Dey R. Pal K. K. Bhatt D. M. Chauhan S. M. (2004). Growth promotion and yield enhancement of peanut (Arachis hypogaea L.) by application of plant growth-promoting rhizobacteria. Microbiol. Res. 159, 371–394. 10.1016/j.micres.2004.08.004 15646384

[B29] Dvorakova M. Hylova A. Soudek P. Retzer K. Spichal L. Vanek T. (2018). Resorcinol-Type Strigolactone Mimics as Potent Germinators of the Parasitic Plants *Striga hermonthica* and *Phelipanche ramosa* . J. Nat. Prod. 81, 2321–2328. 10.1021/acs.jnatprod.8b00160 30362743

[B30] Dvorakova M. Hylova A. Soudek P. Petrova S. Spichal L. Vanek T. (2019). Triazolide strigolactone mimics as potent selective germinators of parasitic plant *Phelipanche ramosa* . Pest Manage. Sci. 75, 2049–2056. 10.1002/ps.5330 30632264

[B31] Ewels P. Magnusson M. Lundin S. Käller M. (2016). MultiQC: summarize analysis results for multiple tools and samples in a single report. Bioinformatics 32, 3047–3048. 10.1093/bioinformatics/btw354 27312411 PMC5039924

[B32] Fitzpatrick C. R. Schneider A. C. (2020). Unique bacterial assembly, composition, and interactions in a parasitic plant and its host. J. Exp. Bot. 71, 2198–2209. 10.1093/jxb/erz572 31912143 PMC7094075

[B33] Fleischmann R. D. Adams M. D. White O. Clayton R. A. Kirkness E. F. Kerlavage A. R. (1995). Whole-genome random sequencing and assembly of Haemophilus influenzae Rd. Science 269, 496–512. 10.1126/science.7542800 7542800

[B34] Flematti G. R. Scaffidi A. Waters M. T. Smith S. M. (2016). Stereospecificity in strigolactone biosynthesis and perception. Planta 243, 1361–1373. 10.1007/s00425-016-2523-5 27105887

[B35] Fu J. Mueller H. de Castro J. V. Yu C. Cavaco-Paulo A. Guebitz G. M. (2011). Changes in the bacterial community structure and diversity during bamboo retting. Biotechnol. J. 6, 1262–1271. 10.1002/biot.201100105 21695788

[B36] Gallart M. Adair K. L. Love J. Meason D. F. Clinton P. W. Xue J. (2018). Host genotype and nitrogen form shape the root microbiome of Pinus radiata. Microb. Ecol. 75, 419–433. 10.1007/s00248-017-1055-2 28875273

[B37] Giraldo A. Crous P. W. (2019). Inside plectosphaerellaceae. Stud. Mycol. 92, 227–286. 10.1016/j.simyco.2018.10.005 30518989 PMC6276054

[B38] Goggin D. E. Emery R. J. N. Kurepin L. V. Powles S. B. (2015). A potential role for endogenous microflora in dormancy release, cytokinin metabolism and the response to fluridone in Lolium rigidum seeds. Ann. Bot. 115, 293–301. 10.1093/aob/mcu231 25471097 PMC4551082

[B39] Goyet V. Billard E. Pouvreau J. B. Lechat M. M. Pelletier S. Bahut M. (2017). Haustorium initiation in the obligate parasitic plant Phelipanche ramosa involves a host-exudated cytokinin signal. J. Exp. Bot. 68, 5539–5552. 10.1093/jxb/erx359 29069455 PMC5853424

[B40] Goyet V. Wada S. Cui S. Wakatake T. Shirasu K. Montiel G. (2019). Haustorium inducing factors for parasitic Orobanchaceae. Front. Plant Sci. 10, 1056. 10.3389/fpls.2019.01056 31555315 PMC6726735

[B41] Großkinsky D. K. Tafner R. Moreno M. V. Stenglein S. A. García de Salamone I. E. Nelson L. M. (2016). Cytokinin production by Pseudomonas fluorescens G20-18 determines biocontrol activity against Pseudomonas syringae in Arabidopsis. Sci. Rep. 6, 23310. 10.1038/srep23310 26984671 PMC4794740

[B42] Hamayun M. Khan S. A. Khan A. L. Rehman G. Kim Y.-H. Iqbal I. (2010). Gibberellin production and plant growth promotion from pure cultures of Cladosporium sp. MH-6 isolated from cucumber (Cucumis sativus L.). Mycologia 102, 989–995. 10.3852/09-261 20943499

[B43] Hardoim P. R. Hardoim C. C. P. van Overbeek L. S. van Elsas J. D. (2012). Dynamics of seed-borne rice endophytes on early plant growth stages. PLoS One 7, e30438. 10.1371/journal.pone.0030438 22363438 PMC3281832

[B44] Hasannejad S. Zad S. J. Alizade H. M. Rahymian H. (2006). The effects of Fusarium oxysporum on broomrape (Orobanche egyptiaca) seed germination. Commun. Agric. Appl. Biol. Sci. 71, 1295–1299. 17390893

[B45] Hayat R. Ahmed I. Sheirdil R. A. (2012). “An overview of plant growth promoting rhizobacteria (PGPR) for sustainable agriculture,” in Crop production for agricultural improvement (Netherlands: Springer), 557–579. 10.1007/978-94-007-4116-4_22

[B46] Henry P. M. Pastrana A. M. Leveau J. H. J. Gordon T. R. (2019). Persistence of Fusarium oxysporum f. Sp. Fragariae in soil through asymptomatic colonization of rotation crops. Phytopathology 109, 770–779. 10.1094/PHYTO-11-18-0418-R 30644330

[B47] Hubbard M. Germida J. Vujanovic V. (2012). Fungal endophytes improve wheat seed germination under heat and drought stress. Botany 90, 137–149. 10.1139/b11-091

[B48] Hussain H. Drogies K.-H. Al-Harrasi A. Hassan Z. Shah A. Rana U. A. (2015). Antimicrobial constituents from endophytic fungus Fusarium sp. Asian Pacific J. Trop. Dis. 5, 186–189. 10.1016/S2222-1808(14)60650-2

[B49] Iasur Kruh L. Lahav T. Abu-Nassar J. Achdari G. Salami R. Freilich S. (2017). Host-parasite-bacteria triangle: the microbiome of the parasitic weed Phelipanche aegyptiaca and tomato-solanum lycopersicum (Mill.) as a host. Front. Plant Sci. 8, 269. 10.3389/fpls.2017.00269 28298918 PMC5331046

[B50] Ishida M. Hara M. Fukino N. Kakizaki T. Morimitsu Y. (2014). Glucosinolate metabolism, functionality and breeding for the improvement of brassicaceae vegetables. Breed. Sci. 64, 48–59. 10.1270/jsbbs.64.48 24987290 PMC4031110

[B51] Joel D. M. Hershenhorn J. Eizenberg H. Aly R. Ejeta G. Rich P. J. (2007). “Biology and management of weedy root parasites,” in Horticultural Reviews (Hoboken, NJ, USA: John Wiley & Sons, Inc), 267–349. 10.1002/9780470168011.ch4

[B52] Johnston-Monje D. Raizada M. N. (2011). Conservation and diversity of seed associated endophytes in Zea across boundaries of evolution, ethnography and ecology. PLoS One 6, e20396. 10.1371/journal.pone.0020396 21673982 PMC3108599

[B53] Kamvar Z. N. Tabima J. F. Grünwald N. J. (2014). Poppr: an R package for genetic analysis of populations with clonal, partially clonal, and/or sexual reproduction. PeerJ 2, e281. 10.7717/peerj.281 24688859 PMC3961149

[B54] Klaedtke S. Jacques M. A. Raggi L. Préveaux A. Bonneau S. Negri V. (2016). Terroir is a key driver of seed-associated microbial assemblages. Environ. Microbiol. 18, 1792–1804. 10.1111/1462-2920.12977 26171841

[B55] Links M. G. Demeke T. Gräfenhan T. Hill J. E. Hemmingsen S. M. Dumonceaux T. J. (2014). Simultaneous profiling of seed-associated bacteria and fungi reveals antagonistic interactions between microorganisms within a shared epiphytic microbiome on Triticum and Brassica seeds. New Phytol. 202, 542–553. 10.1111/nph.12693 24444052 PMC4235306

[B56] Liu J. Yang L. L. Xu C. K. Xi J. Q. Yang F. X. Zhou F. (2012). Sphingobacterium nematocida sp. nov., a nematicidal endophytic bacterium isolated from tobacco. Int. J. Syst. Evol. Microbiol. 62, 1809–1813. 10.1099/ijs.0.033670-0 21984669

[B57] Liu M. Ale M. T. Kołaczkowski B. Fernando D. Daniel G. Meyer A. S. (2017). Comparison of traditional field retting and Phlebia radiata Cel 26 retting of hemp fibres for fibre-reinforced composites. AMB Express 7, 58. 10.1186/s13568-017-0355-8 28275995 PMC5342995

[B58] Lorincz Z. Preininger E. Kósa A. Pónyi T. Nyitrai P. Sarkadi L. (2010). Artificial tripartite symbiosis involving a green alga (Chlamydomonas), a bacterium (Azotobacter) and a fungus (Alternaria): morphological and physiological characterization. Folia Microbiol. (Praha) 55, 393–400. 10.1007/s12223-010-0067-9 20680580

[B59] Louarn J. Carbonne F. Delavault P. Bécard G. Rochange S. (2012). Reduced germination of Orobanche cumana seeds in the presence of arbuscular mycorrhizal fungi or their exudates. PLoS One 7, e49273. 10.1371/journal.pone.0049273 23145139 PMC3492269

[B60] Ma G. Lei L. Xia Z. Gong X. Zhou W. Yang J. (2012). Diversity and phylogenetic analyses of nicotine-degrading bacteria isolated from tobacco plantation soils. Afr. J. Microbiol. Res. 6, 6392–6398. 10.5897/AJMR12.994

[B61] Mašínová T. Bahnmann B. D. Větrovský T. Tomšovský M. Merunková K. Baldrian P. (2017). Drivers of yeast community composition in the litter and soil of a temperate forest. FEMS Microbiol. Ecol. 93, fiw223. 10.1093/femsec/fiw223 27789535

[B62] Mabrouk Y. Zourgui L. Sifi B. Belhadj O. (2007). The potential of Rhizobium strains for biological control of Orobanche crenata. Biol. (Bratisl) 62, 139–143. 10.2478/s11756-007-0021-8

[B63] Machin D. C. Hamon-Josse M. Bennett T. (2020). Fellowship of the rings: a saga of strigolactones and other small signals. New Phytol. 225, 621–636. 10.1111/nph.16135 31442309

[B64] Maida I. Chiellini C. Mengoni A. Bosi E. Firenzuoli F. Fondi M. (2016). Antagonistic interactions between endophytic cultivable bacterial communities isolated from the medicinal plant Echinacea purpurea. Environ. Microbiol. 18, 2357–2365. 10.1111/1462-2920.12911 26013664

[B65] Marín S. Sanchis V. Ramos A. J. Vinas I. Magan N. (1998). Environmental factors, in vitro interactions, and niche overlap between Fusarium moniliforme, F. proliferatum, and F. graminearum, Aspergillus and Penicillium species from maize grain. Mycol. Res. 102, 831–837. 10.1017/S0953756297005777

[B66] Martin M. (2011). Cutadapt removes adapter sequences from high-throughput sequencing reads. EMBnet. J. 17, 10. 10.14806/ej.17.1.200

[B67] Masteling R. Lombard L. de Boer W. Raaijmakers J. M. Dini-Andreote F. (2019). Harnessing the microbiome to control plant parasitic weeds. Curr. Opin. Microbiol. 49, 26–33. 10.1016/j.mib.2019.09.006 31654911 PMC6906922

[B68] Meulenbeld G. H. Hartmans S. (2001). Thioglucosidase activity from Sphingobacterium sp. strain OTG1. Appl. Microbiol. Biotechnol. 56, 700–706. 10.1007/s002530100726 11601617

[B69] Murphy H. Doohan (2018). Endophytic Cladosporium strains from a crop wild relative increase grain yield in barley. Biol. Environ. Proc. R. Irish Acad. 118B, 147. 10.3318/bioe.2018.14

[B70] Nelson P. E. Desjardins A. E. Plattner R. D. (1993). Fumonisins, Mycotoxins Produced by Fusarium Species: Biology, Chemistry, and Significance. Annu. Rev. Phytopathol. 31, 233–252. 10.1146/annurev.py.31.090193.001313 18643768

[B71] Nelson P. E. Dignani M. C. Anaissie E. J. (1994). Taxonomy, biology, and clinical aspects of Fusarium species. Clin. Microbiol. Rev. 7, 479–504. 10.1128/CMR.7.4.479 7834602 PMC358338

[B72] Nelson E. B. (2018). The seed microbiome: origins, interactions, and impacts. Plant Soil. 422, 7–34. 10.1007/s11104-017-3289-7

[B73] Nilsson R. H. Larsson K.-H. Taylor A. F. S. Bengtsson-Palme J. Jeppesen T. S. Schigel D. (2019). The UNITE database for molecular identification of fungi: handling dark taxa and parallel taxonomic classifications. Nucleic Acids Res. 47, 259–264. 10.1093/nar/gky1022 PMC6324048 30371820

[B74] Noor A. II Nava A. Cooke P. Cook D. Creamer R. (2018). Evidence for nonpathogenic relationships of Alternaria section Undifilum endophytes within three host locoweed plant species. Botany 96, 187–200. 10.1139/cjb-2017-0117

[B75] Ohtsuru M. Tsuruo I. Hata T. (1969). Studies on Fungous myrosinase. Agric. Biol. Chem. 33, 1309–1325. 10.1080/00021369.1969.10859462

[B76] Oteino N. Lally R. D. Kiwanuka S. Lloyd A. Ryan D. Germaine K. J. (2015). Plant growth promotion induced by phosphate solubilizing endophytic Pseudomonas isolates. Front. Microbiol. 6, 745. 10.3389/fmicb.2015.00745 26257721 PMC4510416

[B77] Palm M. E. Rotem J. (1997). The Genus alternaria: biology, epidemiology, and pathogenicity. Mycologia 89, 347. 10.2307/3761094

[B78] Pappas M. L. Liapoura M. Papantoniou D. Avramidou M. Kavroulakis N. Weinhold A. (2018). The beneficial endophytic fungus fusariumsolani strain k alters tomato responses against spider mites to the benefit of the plant. Front. Plant Sci. 9, 1603. 10.3389/fpls.2018.01603 PMC6232530 30459791

[B79] Parker C. Riches C. R. (1993). Parasitic weeds of the world: biology and control (Wallingford: CAB International).

[B80] Parker C. (2012). Parasitic weeds: a world challenge. Weed Sci. 60, 269–276. 10.1614/WS-D-11-00068.1

[B81] Patten C. L. Glick B. R. (2002). Role of Pseudomonas putida indoleacetic acid in development of the host plant root system. Appl. Environ. Microbiol. 68, 3795–3801. 10.1128/AEM.68.8.3795-3801.2002 12147474 PMC124051

[B82] Perelló A. E. Larrán S. (2013). Nature and effect of Alternaria spp. complex from wheat grain on germination and disease transmission. Pak. J. Bot. 45, 1817–1824.

[B83] Pouvreau J.-B. Gaudin Z. Auger B. Lechat M.-M. Gauthier M. Delavault P. (2013). A high-throughput seed germination assay for root parasitic plants. Plant Methods 9, 32. 10.1186/1746-4811-9-32 23915294 PMC3751143

[B84] Puente M. E. Li C. Y. Bashan Y. (2009). Endophytic bacteria in cacti seeds can improve the development of cactus seedlings. Environ. Exp. Bot. 66, 402–408. 10.1016/j.envexpbot.2009.04.007

[B85] Quast C. Pruesse E. Yilmaz P. Gerken J. Schweer T. Yarza P. (2012). The SILVA ribosomal RNA gene database project: improved data processing and web-based tools. Nucleic Acids Res. 41, D590–D596. 10.1093/nar/gks1219 23193283 PMC3531112

[B86] Raimondo M. L. Carlucci A. (2018). Characterization and pathogenicity assessment of Plectosphaerella species associated with stunting disease on tomato and pepper crops in Italy. Plant Pathol. 67, 626–641. 10.1111/ppa.12766

[B87] Revell L. J. (2012). phytools: an R package for phylogenetic comparative biology (and other things). Methods Ecol. Evol. 3, 217–223. 10.1111/j.2041-210X.2011.00169.x

[B88] Rezki S. Campion C. Iacomi-Vasilescu B. Preveaux A. Toualbia Y. Bonneau S. (2016). Differences in stability of seed-associated microbial assemblages in response to invasion by phytopathogenic microorganisms. PeerJ 4, e1923. 10.7717/peerj.1923 27077013 PMC4830237

[B89] Ribeiro A. Pochart P. Day A. Mennuni S. Bono P. Baret J. L. (2015). Microbial diversity observed during hemp retting. Appl. Microbiol. Biotechnol. 99, 4471–4484. 10.1007/s00253-014-6356-5 25575888

[B90] Ritz C. Baty F. Streibig J. C. Gerhard D. (2015). Dose-Response Analysis Using R. PLoS One 10, e0146021. 10.1371/journal.pone.0146021 26717316 PMC4696819

[B91] Robin A. H. K. Yi G. E. Laila R. Hossain M. R. Park J. II Kim H. R. (2017). Leptosphaeria maculans alters glucosinolate profiles in blackleg disease–resistant and -susceptible cabbage lines. Front. Plant Sci. 8, 1769. 10.3389/fpls.2017.01769 29075281 PMC5644266

[B92] Romeo L. Iori R. Rollin P. Bramanti P. Mazzon E. (2018). Isothiocyanates: an overview of their antimicrobial activity against human infections. Molecules 23, 624. 10.3390/molecules23030624 PMC6017699 29522501

[B93] Rosenblueth M. Martínez-Romero E. (2006). Bacterial endophytes and their interactions with hosts. Mol. Plant-Microbe Interact. 19, 827–837. 10.1094/MPMI-19-0827 16903349

[B94] Ryan R. P. Monchy S. Cardinale M. Taghavi S. Crossman L. Avison M. B. (2009). The versatility and adaptation of bacteria from the genus Stenotrophomonas. Nat. Rev. Microbiol. 7, 514–525. 10.1038/nrmicro2163 19528958

[B95] Sørensen J. Nybroe O. (2004). “Pseudomonas in the soil environment,” in Pseudomonas (Boston, MA: Springer US), 369–401. 10.1007/978-1-4419-9086-0_12

[B96] Salter S. J. Cox M. J. Turek E. M. Calus S. T. Cookson W. O. Moffatt M. F. (2014). Reagent and laboratory contamination can critically impact sequence-based microbiome analyses. BMC Biol. 12, 87. 10.1186/s12915-014-0087-z 25387460 PMC4228153

[B97] Sapkota R. Knorr K. Jørgensen L. N. O’Hanlon K. A. Nicolaisen M. (2015). Host genotype is an important determinant of the cereal phyllosphere mycobiome. New Phytol. 207, 1134–1144. 10.1111/nph.13418 25898906

[B98] Scaffidi A. Waters M. T. Sun Y. K. Skelton B. W. Dixon K. W. Ghisalberti E. L. (2014). Strigolactone hormones and their stereoisomers signal through two related receptor proteins to induce different physiological responses in Arabidopsis. Plant Physiol. 165, 1221–1232. 10.1104/pp.114.240036 24808100 PMC4081333

[B99] Schulz B. Haas S. Junker C. Andrée N. Schobert M. (2015). Fungal endophytes are involved in multiple balanced antagonisms. Curr. Sci. 109, 39–45.

[B100] Screpanti C. Yoneyama K. Bouwmeester H. J. (2016). Strigolactones and parasitic weed management 50 years after the discovery of the first natural strigolactone strigol: status and outlook. Pest Manage. Sci. 72, 2013–2015. 10.1002/ps.4436 27700003

[B101] Shade A. Jacques M.-A. Barret M. (2017). Ecological patterns of seed microbiome diversity, transmission, and assembly. Curr. Opin. Microbiol. 37, 15–22. 10.1016/J.MIB.2017.03.010 28437661

[B102] Smith B. J. Kirkegaard J. A. (2002). In vitro inhibition of soil microorganisms by 2-phenylethyl isothiocyanate. Plant Pathol. 51, 585–593. 10.1046/j.1365-3059.2002.00744.x

[B103] Smith S. N. (2007). An overview of ecological and habitat aspects in the genus Fusarium with special emphasis on the soil-borne pathogenic forms. Plant Pathol. Bull. 16, 97–120.

[B104] Steyn P. L. Segers P. Vancanneyt M. Sandra P. Kersters K. Joubert J. J. (1998). Classification of heparinolytic bacteria into a new genus, Pedobacter, comprising four species: Pedobacter heparinus comb. nov., Pedobacter piscium comb. nov., Pedobacter africanus sp. nov. and Pedobacter saltans sp. nov. proposal of the family Sphingobac. Int. J. Syst. Bacteriol. 48, 165–177. 10.1099/00207713-48-1-165 9542086

[B105] Stojanova B. Delourme R. Duffé P. Delavault P. Simier P. (2019). Genetic differentiation and host preference reveal non-exclusive host races in the generalist parasitic weed Phelipanche ramosa. Weed Res. 59, 107–118. 10.1111/wre.12353

[B106] Szűcs Z. Plaszkó T. Cziáky Z. Kiss-Szikszai A. Emri T. Bertóti R. (2018). Endophytic fungi from the roots of horseradish (Armoracia rusticana) and their interactions with the defensive metabolites of the glucosinolate - myrosinase - isothiocyanate system. BMC Plant Biol. 18, 85. 10.1186/s12870-018-1295-4 29743024 PMC5944135

[B107] Thomma B. P. H. J. (2003). Alternaria spp.: from general saprophyte to specific parasite. Mol. Plant Pathol. 4, 225–236. 10.1046/j.1364-3703.2003.00173.x 20569383

[B108] Toh S. Holbrook-Smith D. Stogios P. J. Onopriyenko O. Lumba S. Tsuchiya Y. (2015). Structure-function analysis identifies highly sensitive strigolactone receptors in Striga. Science 350, 203–207. 10.1126/science.aac9476 26450211

[B109] Tsialtas J. T. Eleftherohorinos I. G. (2011). First report of branched broomrape (Orobanche ramosa) on Oilseed Rape (Brassica napus), Wild mustard (Sinapis arvensis), and wild vetch (Vicia spp.) in Northern Greece. Plant Dis. 95, 1322–1322. 10.1094/PDIS-06-11-0462 30731672

[B110] Vaz Patto M. C. FernÁndez-Aparicio M. Satovic Z. Rubiales D. (2009). Extent and pattern of genetic differentiation within and between European populations of Phelipanche ramosa revealed by amplified fragment length polymorphism analysis. Weed Res. 49, 48–55. 10.1111/j.1365-3180.2009.00740.x

[B111] Verma S. K. White J. F. (Eds.) (2019). Seed Endophytes (Cham: Springer International Publishing). 10.1007/978-3-030-10504-4

[B112] Walitang D. II Kim C.-G. Kim K. Kang Y. Kim Y. K. Sa T. (2018). The influence of host genotype and salt stress on the seed endophytic community of salt-sensitive and salt-tolerant rice cultivars. BMC Plant Biol. 18, 51. 10.1186/s12870-018-1261-1 29587643 PMC5870378

[B113] Welte C. U. Rosengarten J. F. de Graaf R. M. Jetten M. S. M. (2016). SaxA-mediated isothiocyanate metabolism in phytopathogenic pectobacteria. Appl. Environ. Microbiol. 82, 2372–2379. 10.1128/AEM.04054-15 26873319 PMC4959473

[B114] Westwood J. H. (2013). “The physiology of the established parasite–host association,” in Parasitic Orobanchaceae (Berlin, Heidelberg: Springer Berlin Heidelberg), 87–114. 10.1007/978-3-642-38146-1_6

[B115] Xie X. Yoneyama K. Kisugi T. Uchida K. Ito S. Akiyama K. (2013). Confirming stereochemical structures of strigolactones produced by rice and tobacco. Mol. Plant 6, 153–163. 10.1093/mp/sss139 23204500 PMC3548624

[B116] Xie X. (2016). Structural diversity of strigolactones and their distribution in the plant kingdom. J. Pestic. Sci. 41, 175–180. 10.1584/jpestics.J16-02 30363158 PMC6140701

[B117] Xu D. Hanschen F. S. Witzel K. Nintemann S. J. Nour-Eldin H. H. Schreiner M. (2017). Rhizosecretion of stele-synthesized glucosinolates and their catabolites requires GTR-mediated import in Arabidopsis. J. Exp. Bot. 68, 3205–3214. 10.1093/jxb/erw355 27702989 PMC5853541

[B118] Yoneyama K. K. Awad A. A. Xie X. Yoneyama K. K. Takeuchi Y. (2010). Strigolactones as germination stimulants for root parasitic plants. Plant Cell Physiol. 51, 1095–1103. 10.1093/pcp/pcq055 20403809 PMC2900819

[B119] Yoneyama K. Xie X. Yoneyama K. Kisugi T. Nomura T. Nakatani Y. (2018). Which are the major players, canonical or non-canonical strigolactones? J. Exp. Bot. 69, 2231–2239. 10.1093/jxb/ery090 29522151

[B120] Zhou X. K. Li Q. Q. Mo M. H. Zhang Y. G. Dong L. M. Xiao M. (2017). Sphingobacterium tabacisoli sp. Nov., isolated from a tobacco field soil sample. Int. J. Syst. Evol. Microbiol. 67, 4808–4813. 10.1099/ijsem.0.002381 28984223

