# Supplementary material for: Populations of the Parasitic Plant Phelipanche ramosa Influence Their Seed Microbiota
Source: Front Plant Sci. 2020 Jul 17;11:1075. doi: 10.3389/fpls.2020.01075 (PMC7379870; doi:10.3389/fpls.2020.01075)
Supplement: Supplementary file 1 [file DataSheet_1.pdf]

Supplemental Figures and Tables

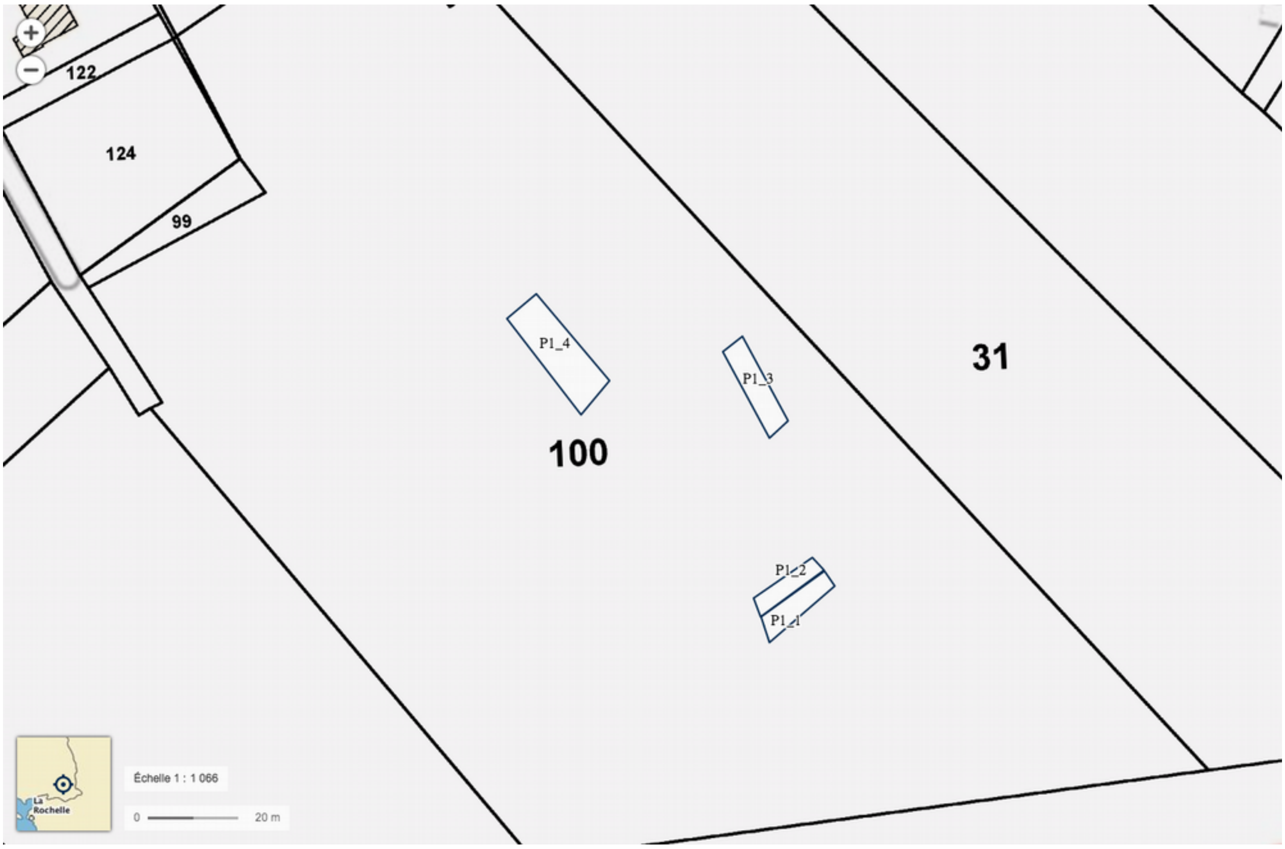

A

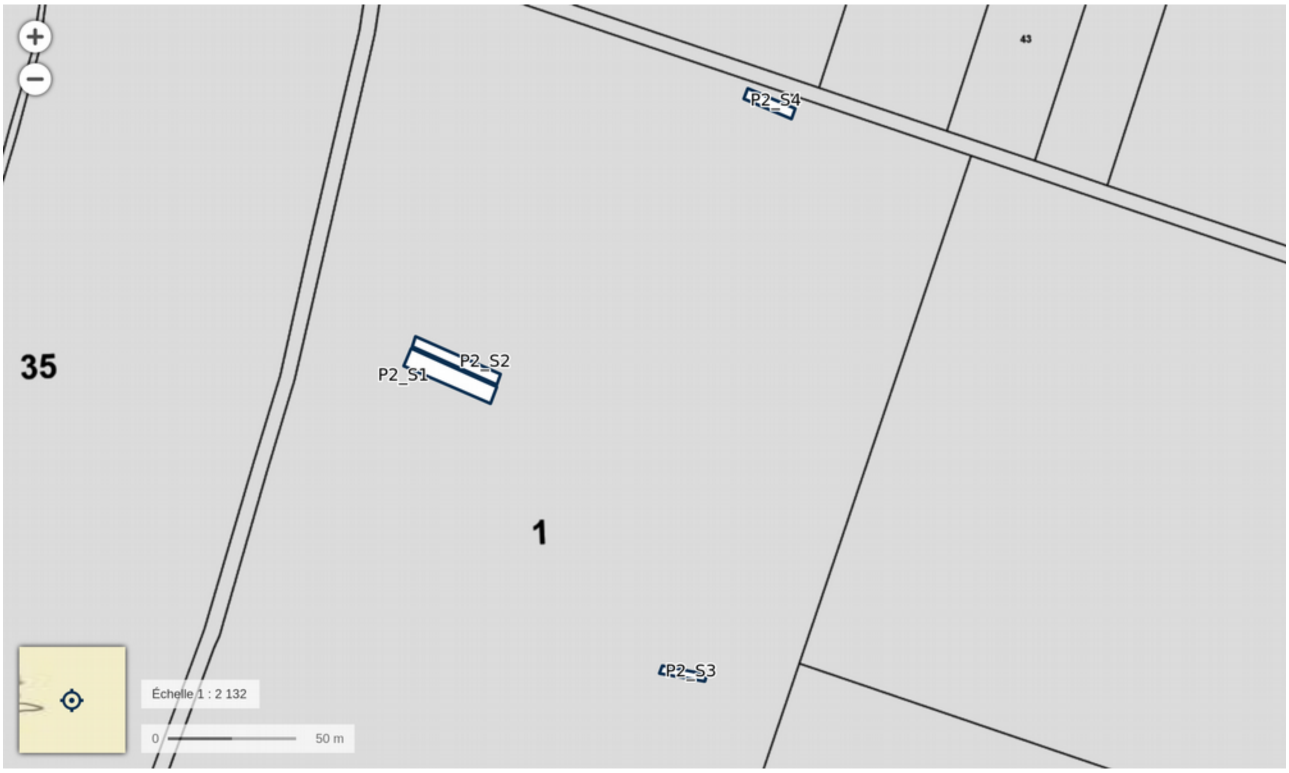

B

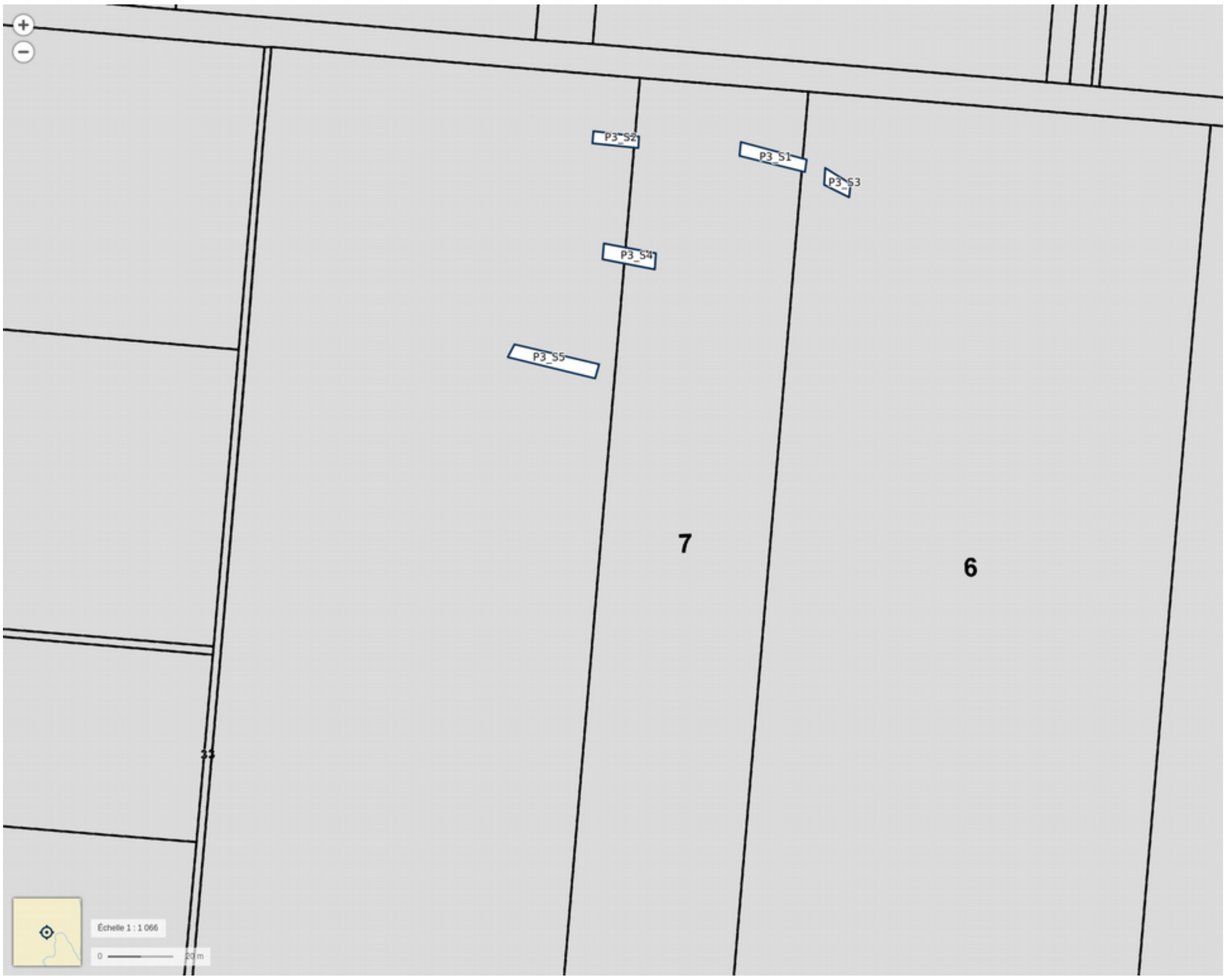
P3_S2
P3_S1
P3_S3
P3_S4
P3_S5
7
6
Échelle 1 : 1 066
C

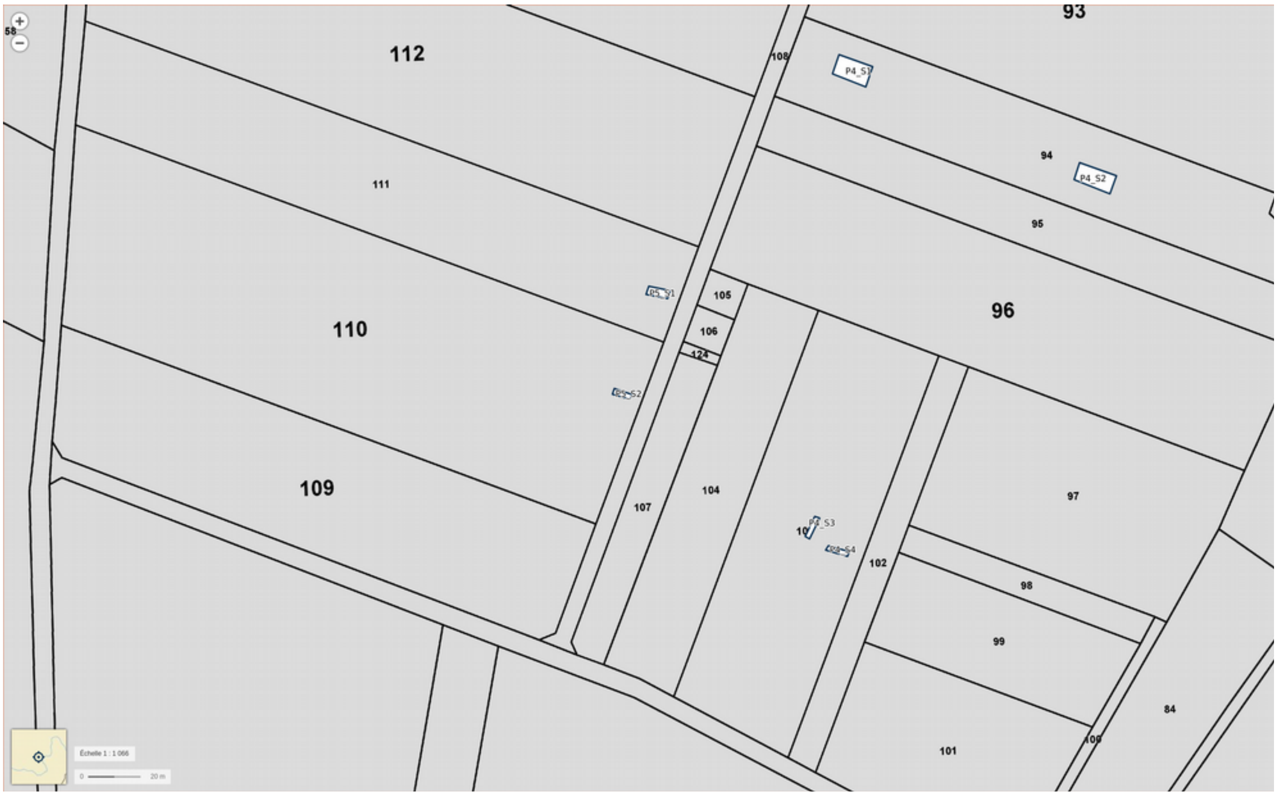
112
93
108
P4_S1
111
94
P4_S2
95
105
106
124
110
96
109
107
104
97
102
98
99
84
101
Échelle 1 : 1 066
D

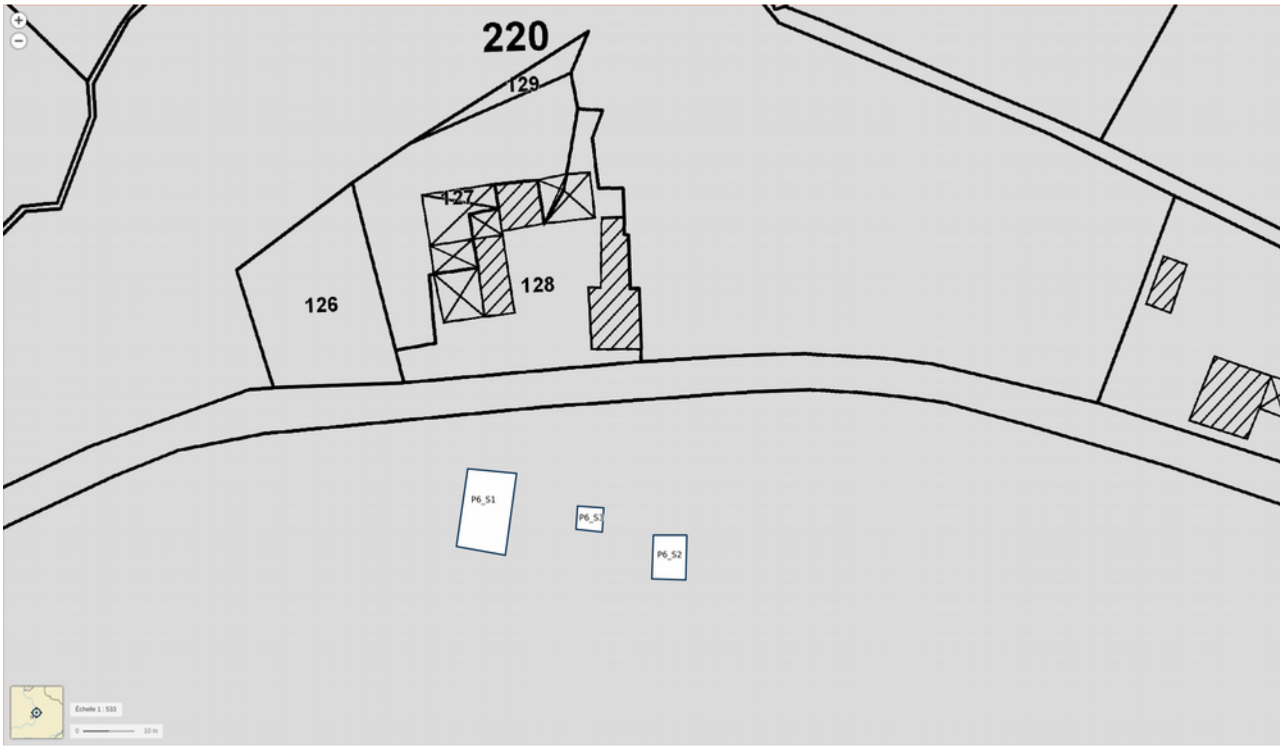

E

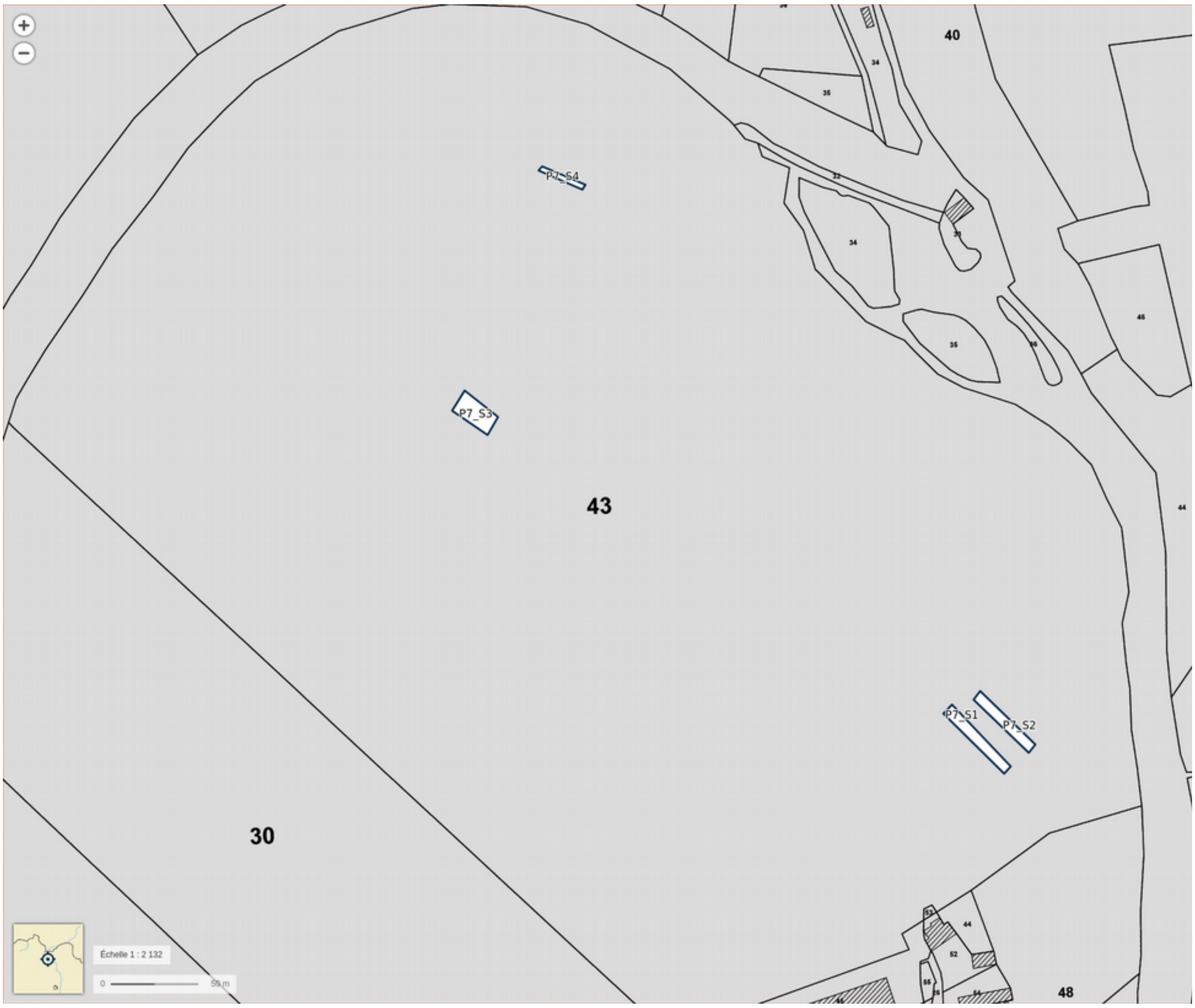

F

Figure S1 Geoplots of sampled area for each sample in the different plots : A-P1, B-P2,C- P3, D-P4 & P5, E-P6 and F-P7 from Geoportail

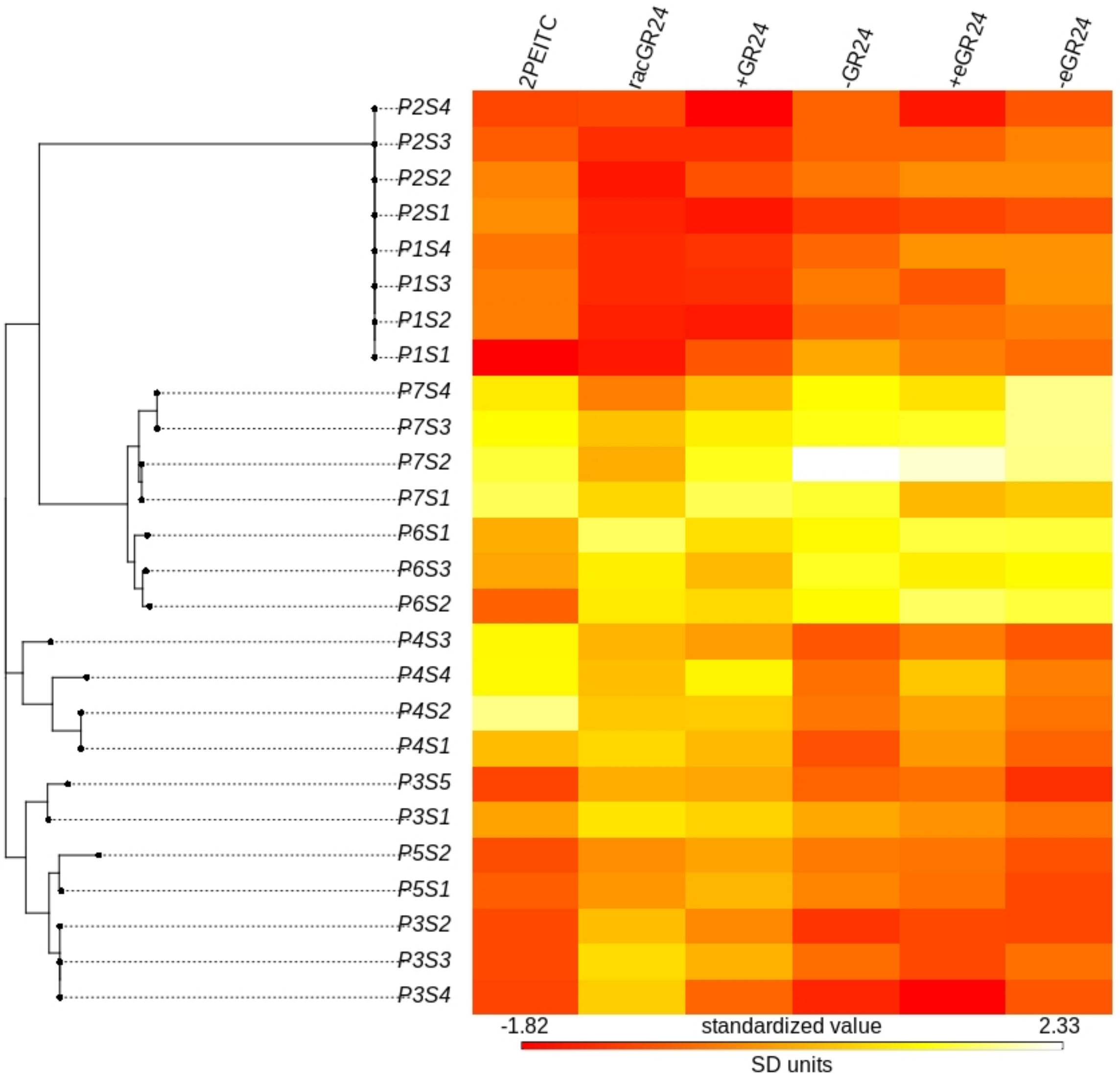

Figure S2 Phylogenetic tree with standardized GS sensitivity heatmap from log10(EC50). Legend: Red = highly GS sensitive, white = moderately GS sensitive

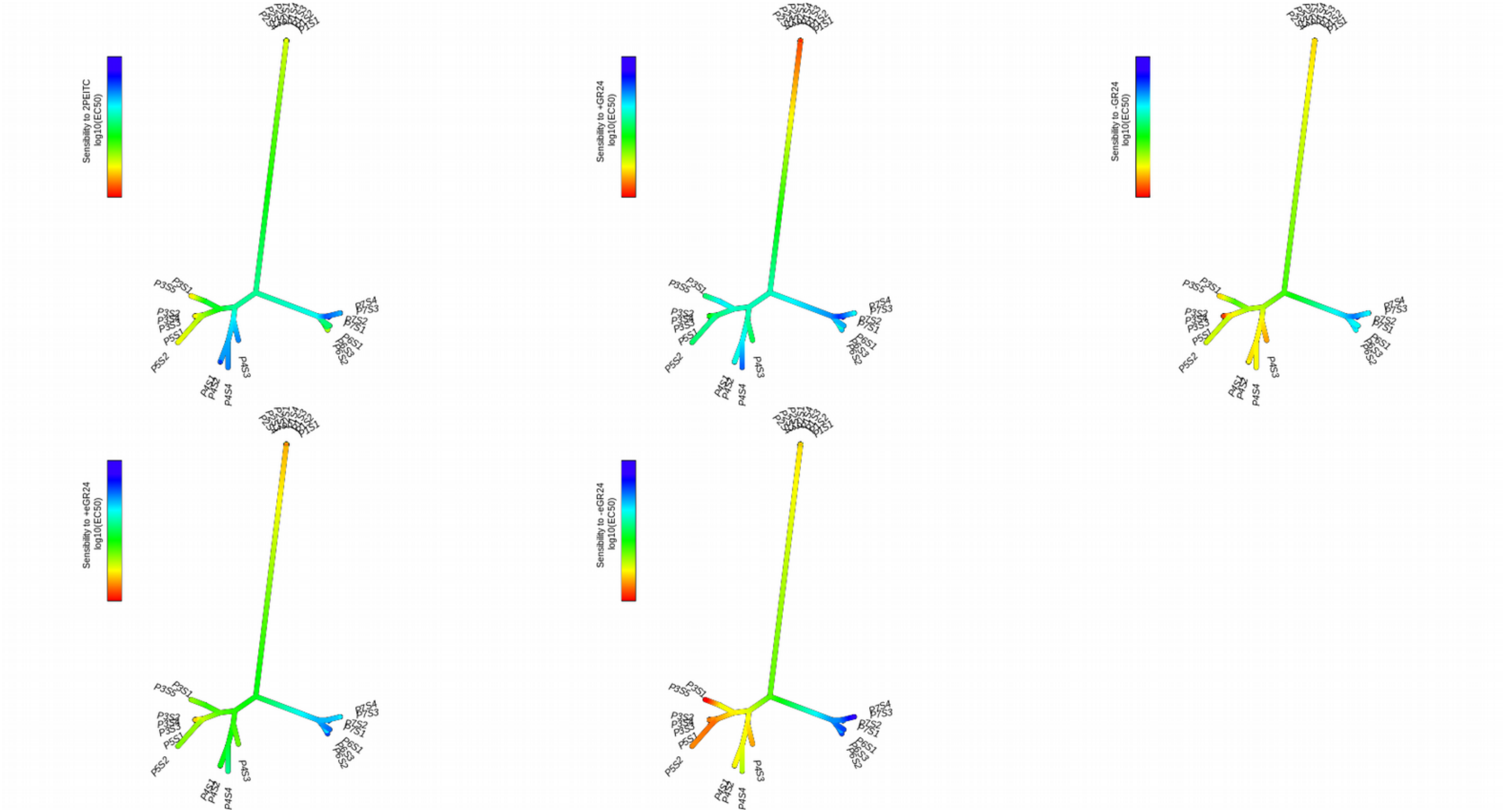

Figure S3 Phylogenetic tree with GS sensitivity inferred from log10(EC50) seed sample values A. 2PEITC B. racGR24 :(-)+GR24 & (-)-GR24, C. (-)+GR24 , D. (+)-GR24, E (+)+eGR24, F. (+)-eGR24. Legend : Red = highly GS sensitive, blue = moderately GS sensitive

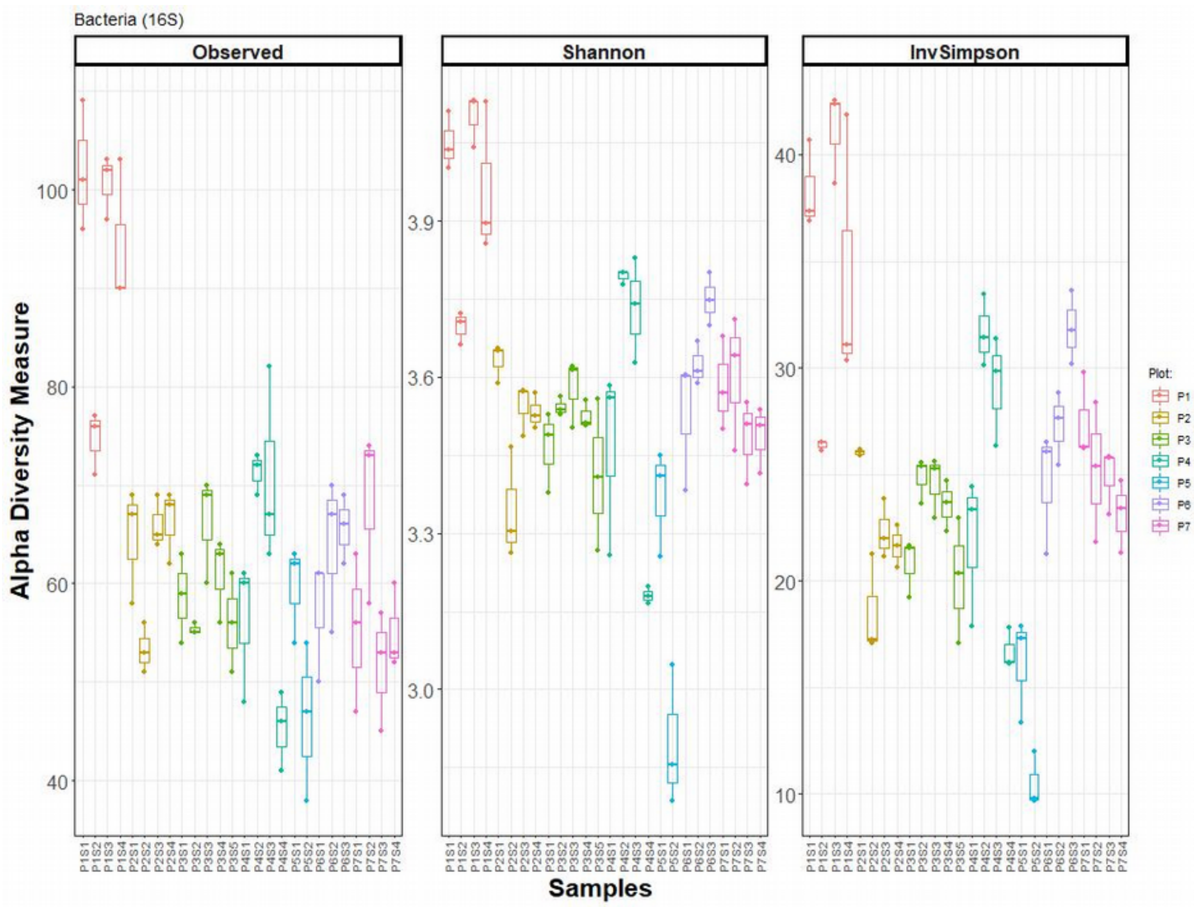

A.

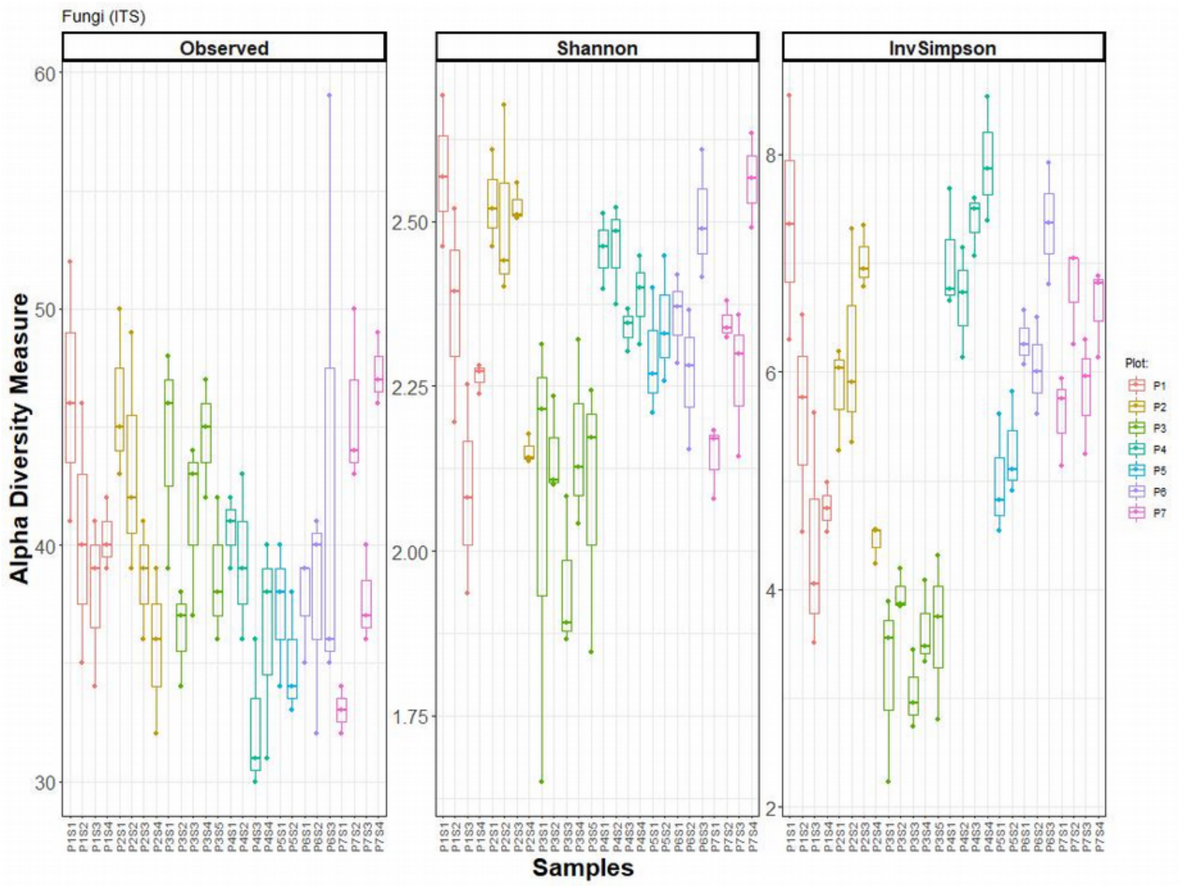

B.

Figure S4 Alpha diversity of A. bacterial and B. fungal seed communities estimated as the Observed richness, Simpson index, and Inverse Shannon index on 3 biological replicates for each sample.

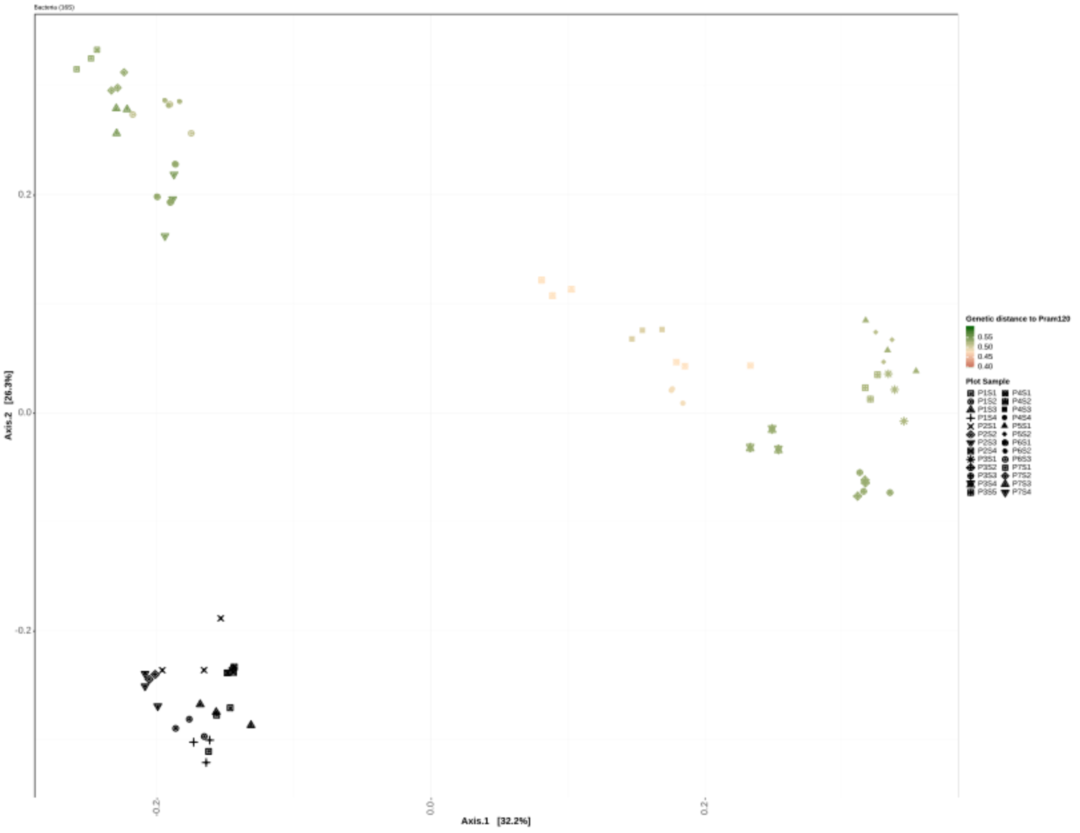

A

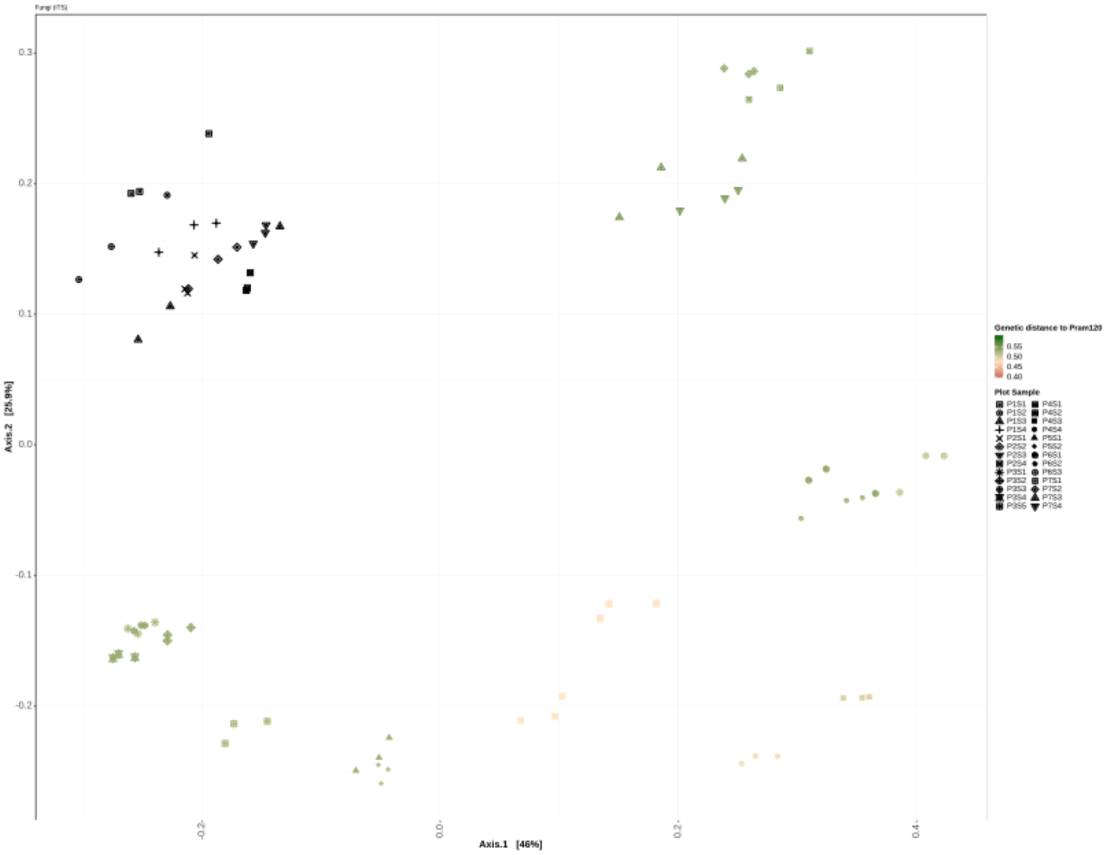

B

Figure S5 PCoA of 16S seed profile (A) and ITS seed profile (B) with respect to genetic distance to oilseed rape *P. ramosa* Pram120 reference lot (as color gradient, black refers to nule genetic distance to Pram120) and plot Sample (shapes)

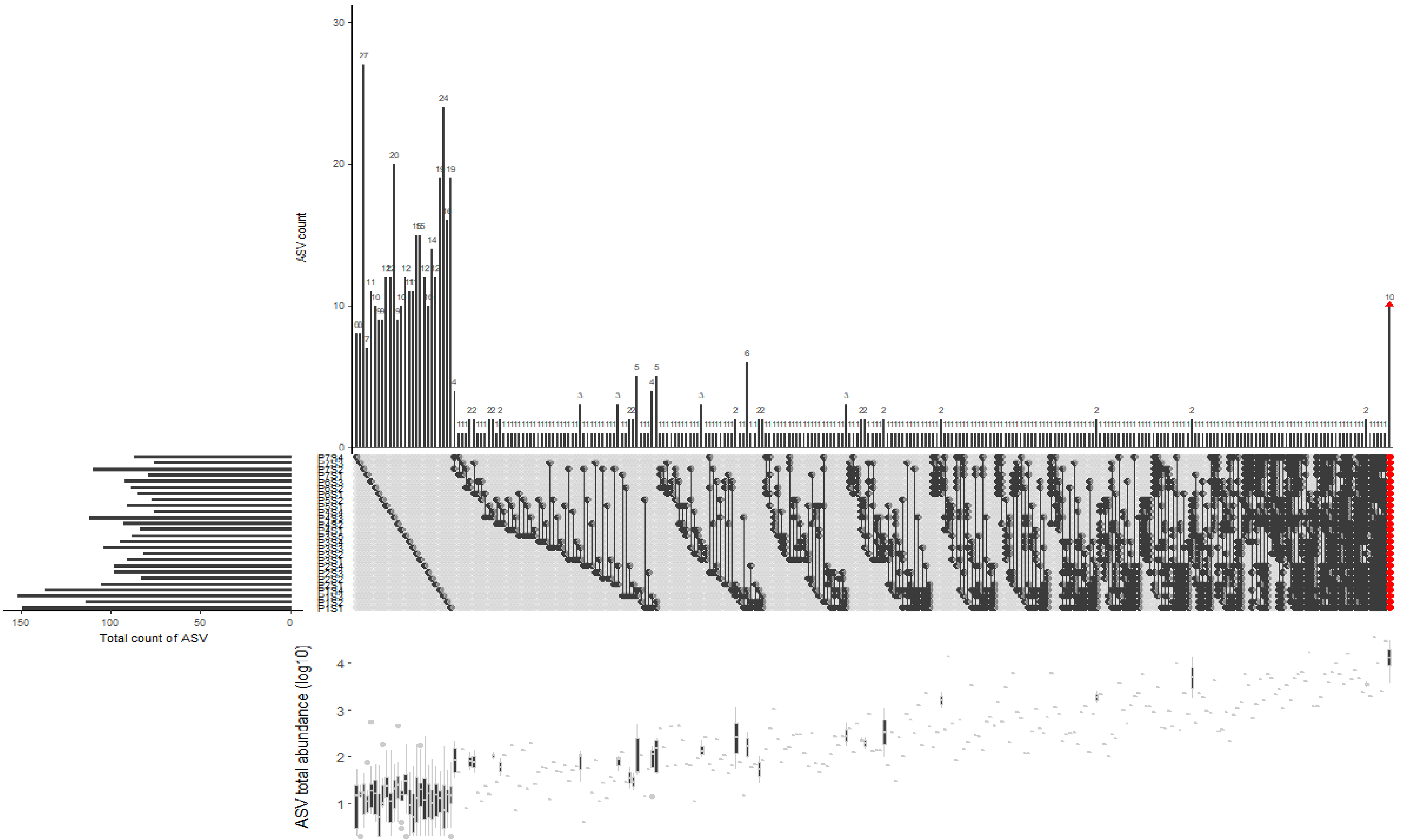

Figure S6 Upset graph of unique and shared 16S ASVs between all seed samples and their average abundances (log10)

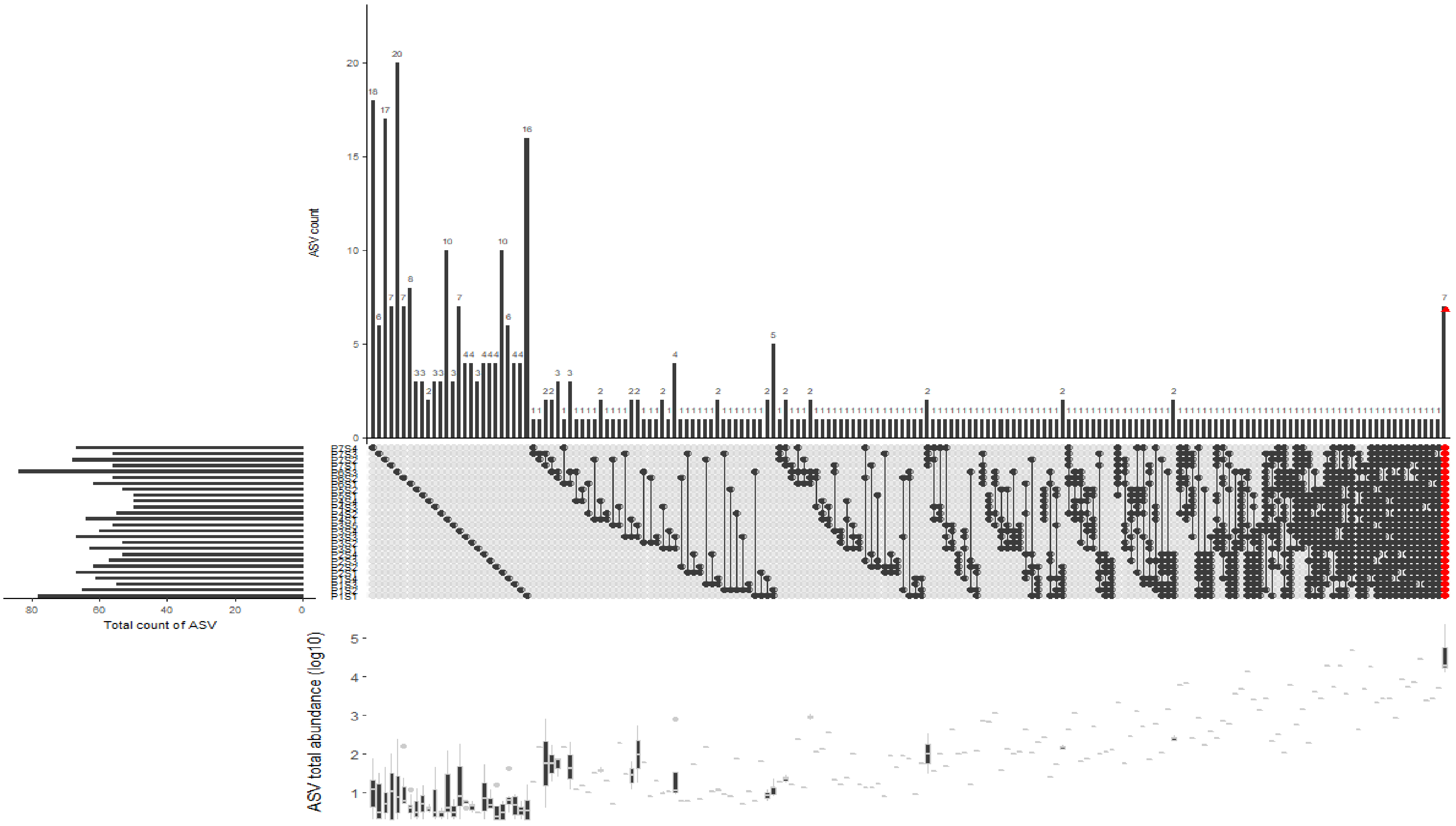

Figure S7 Upset graph of unique and shared ITS ASVs between all seed samples and their average abundances (log10)

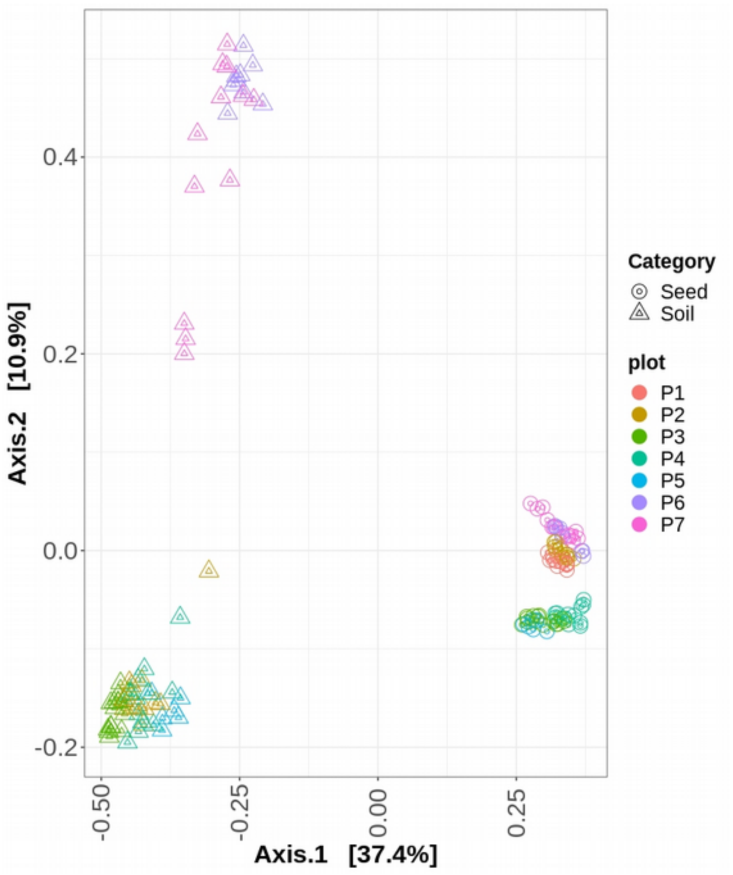

A

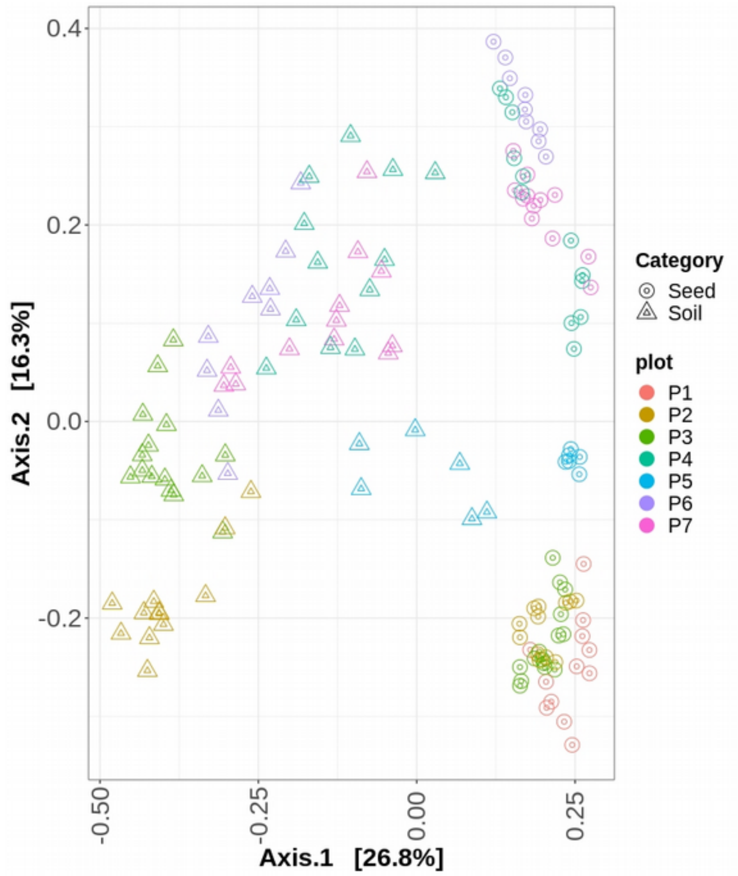

B

Figure S8 PCoA analysis of seed A. 16S and B. ITS1 communities based on Bray-Curtis index on seed and soil samples according to sampling plots (in color)

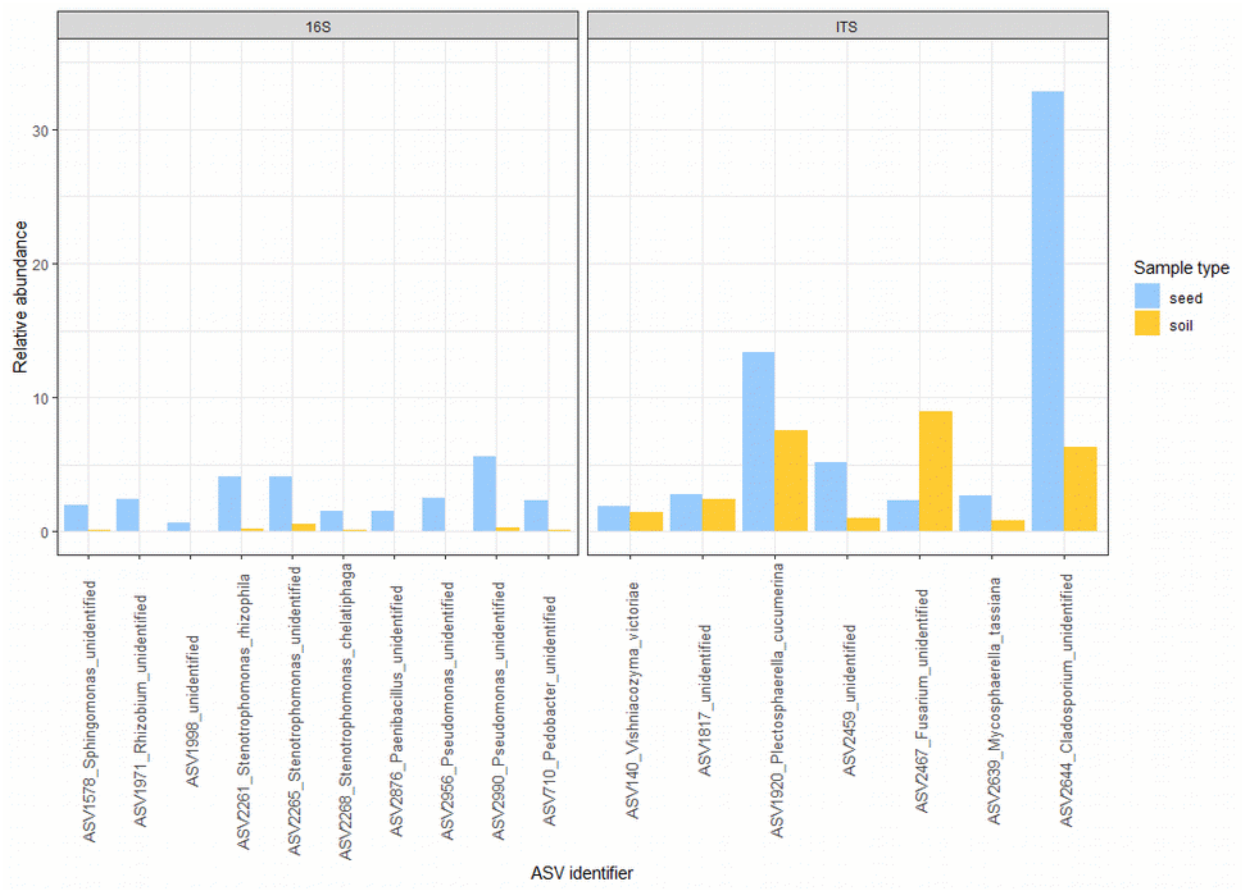

A

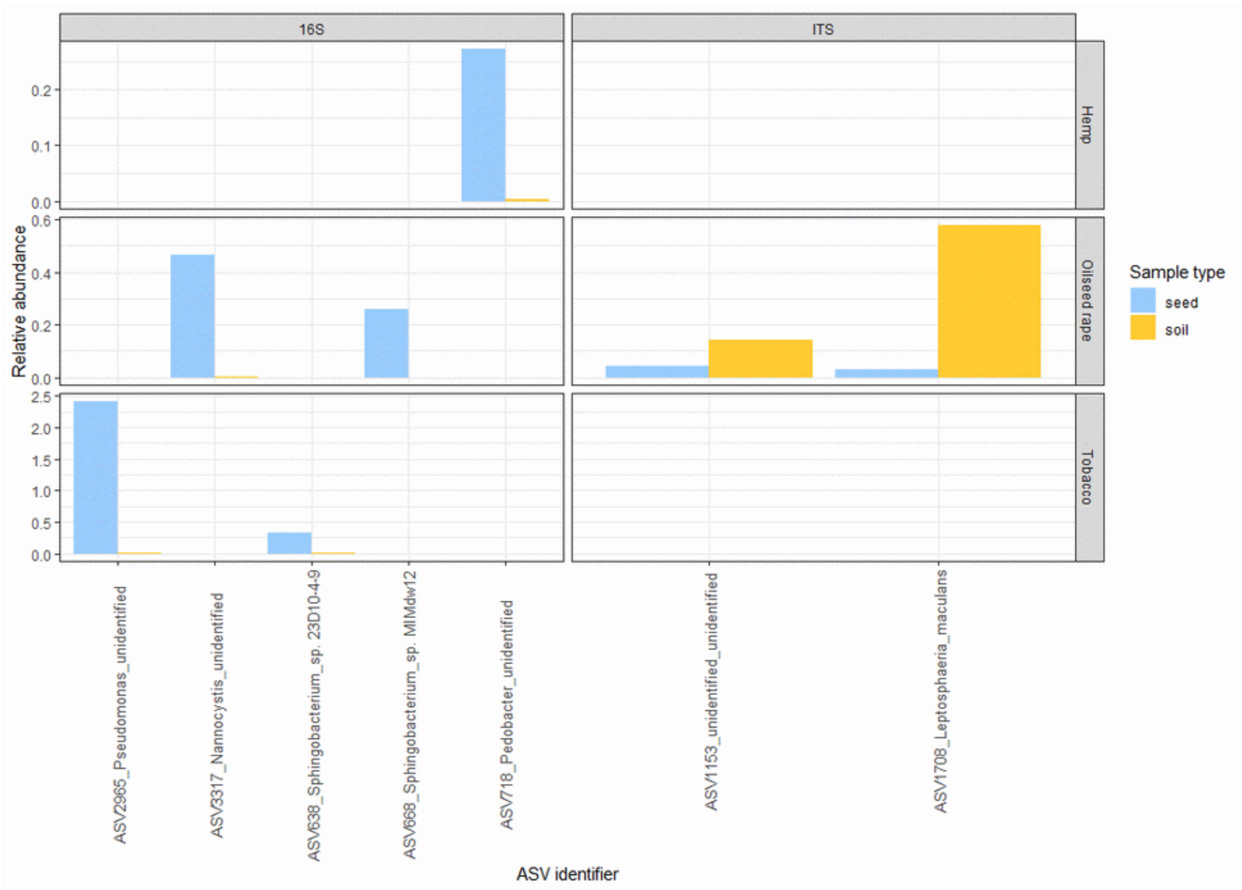

B

Figure S9 Relative abundance of A. all seed core ASVs and B. host-related seed core ASVs in seeds (blue) and corresponding soils (yellow)

Table S1 Characteristics of samples

Sample	Plot	2017 crop	Municipality	Latitude	Longitude	Eco-region	Sampling date
**P1S1**	P1	Oilseed rape	Fontenay-le-compte	46.445115	-0.767713	F12	10/07/17
**P1S2**	P1	Oilseed rape	Fontenay-le-compte	46.445556	-0.767656	F12	10/07/17
**P1S3**	P1	Oilseed rape	Fontenay-le-compte	46.445518	-0.767708	F12	10/07/17
**P1S4**	P1	Oilseed rape	Fontenay-le-compte	46.445567	-0.76825	F12	10/07/17
**P2S1**	P2	Oilseed rape	Saint Ouenne	46.430524	-0.4671	F12	10/07/17
**P2S2**	P2	Oilseed rape	Saint Ouenne	46.430495	-0.466671	F12	10/07/17
**P2S3**	P2	Oilseed rape	Saint Ouenne	46.429393	-0.465865	F12	10/07/17
**P2S4**	P2	Oilseed rape	Saint Ouenne	46.431438	-0.46541	F12	10/07/17
**P3S1**	P3	Tobacco	Pliboux, le Grand Cerzé	46.17542	0.104627	F12	14/09/17
**P3S2**	P3	Tobacco	Pliboux, le Grand Cerzé	46.175537	0.104187	F11	14/09/17
**P3S3**	P3	Tobacco	Pliboux, le Grand Cerzé	46.175418	0.104516	F11	14/09/17
**P3S4**	P3	Tobacco	Pliboux, le Grand Cerzé	46.175532	0.103621	F11	14/09/17
**P3S5**	P3	Tobacco	Pliboux, le Grand Cerzé	46.174941	0.10326	F11	14/09/17
**P4S1**	P4	Tobacco	Aigre, Crève Cœur	45.884015	-0.00879	F12	14/09/17
**P4S2**	P4	Tobacco	Aigre, Crève Cœur	45.884396	-0.009975	F12	14/09/17
**P4S3**	P4	Tobacco	Aigre, Crève Cœur	45.882836	-0.010193	F12	14/09/17
**P4S4**	P4	Tobacco	Aigre, Crève Cœur	45.88275	-0.010003	F12	14/09/17
**P5S1**	P5	Tobacco	Aigre, Crève Cœur	45.883654	-0.01087	F12	14/09/17
**P5S2**	P5	Tobacco	Aigre, Crève Cœur	45.883301	-0.011136	F12	14/09/17
**P6S1**	P6	Hemp	Neuville-sur-Sarthe, Beaulieu	48.069946	0.213361	B61	25/09/17
**P6S2**	P6	Hemp	Neuville-sur-Sarthe, Beaulieu	48.069753	0.213846	B61	25/09/17
**P6S3**	P6	Hemp	Neuville-sur-Sarthe, Beaulieu	48.06985	0.213652	B61	25/09/17
**P7S1**	P7	Hemp	Assé le boisne, Chanteloup	48.301193	-0.006227	A13	25/09/17
**P7S2**	P7	Hemp	Assé le boisne, Chanteloup	48.301217	-0.00614	A13	25/09/17
**P7S3**	P7	Hemp	Assé le boisne, Chanteloup	48.304185	-0.009447	A13	25/09/17
**P7S4**	P7	Hemp	Assé le boisne, Chanteloup	48.304277	-0.009784	A13	25/09/17

Table S2 Ten year-history of crop rotation in sampled fields based on Geoportail graphical plot register: crop areas declared by farmers from 2007-2017.

Year	P1	P2	P3	P4	P5	P6	P7
**2017**	**WOSR**	**WOSR**	**Tobacco**	**Tobacco**	**Tobacco**	**Hemp**	**Hemp**
**2016**	Winter soft wheat	Sunflower	**Tobacco**	**Tobacco**	**Tobacco**	**Hemp**	Winter Barley
**2015**	Winter Durum wheat	WOSR	**Tobacco**	**Tobacco**	**Tobacco**	**Hemp**	Winter soft wheat
**2014**	**WOSR**	Soft wheat	Sunflower	Sunflower	Maize	Barley	Winter soft wheat
**2013**	Durum wheat	Sunflower	Soft wheat	Maize	Maize	Maize	Maize
**2012**	Other oleaginous	Sunflower	Maize	Maize	Maize	Cereals	Barley
**2011**	Soft wheat	Soft wheat	Soft wheat	Maize	Tobacco	Cereals	Winter soft wheat
**2010**	**WOSR**	**WOSR**	Sunflower	Maize	Sunflower	Maize	Protein crops
**2009**	Winter Barley	Sunflower	Soft wheat	Maize	Maize	Cereals	Protein crops
**2008**	Sunflower	Soft wheat	Sunflower	Maize	Cereals	Cereals	Meadow
**2007**	Soft wheat	**WOSR**	Soft wheat	Maize	Maize	Maize	Winter soft wheat

Table S3 Amplicon sizes of the twenty SSR markers on the 26 seed samples

Sample	PR-ccTri01		PR-nDi17		Phera19		Phera28		Phera38		PR-nDi02		PR-cDi01		Phera10		PR-nTri02		PR-cTri11		Phera02		PR-nTri08		PR-cTri03		PR-cDi09		Phera40		PR-nDi01		PR-cDi05		Phera18		Phera20		Phera46	
**P1S1**	125	125	143	143	162	162	216	216	278	278	179	179	217	217	136	136	265	265	218	218	136	136	255	255	119	119	153	162	189	189	200	200	224	224	150	150	177	177	277	277
**P1S2**	125	125	143	143	162	162	216	216	278	278	179	179	217	217	136	136	265	265	218	218	136	136	255	255	119	119	153	162	189	189	200	200	224	224	150	150	177	177	277	277
**P1S3**	125	125	143	143	162	162	216	216	278	278	179	179	217	217	136	136	265	265	218	218	136	136	255	255	119	119	153	162	189	189	200	200	224	224	150	150	176	176	277	277
**P1S4**	125	125	143	143	162	162	216	216	278	278	179	179	217	217	136	136	265	265	218	218	136	136	255	255	119	119	153	162	189	189	200	200	224	224	150	150	177	177	277	277
**P2S1**	125	125	143	143	162	162	216	216	278	278	179	179	217	217	136	136	265	265	218	218	135	135	255	255	119	119	153	162	188	188	200	200	224	224	150	150	177	177	277	277
**P2S2**	125	125	143	143	162	162	216	216	278	278	179	179	217	217	136	136	265	265	218	218	136	136	255	255	119	119	153	162	189	189	200	200	224	224	150	150	177	177	277	277
**P2S3**	125	125	143	143	162	162	216	216	278	278	179	179	217	217	136	136	265	265	218	218	136	136	255	255	119	119	153	162	189	189	200	200	224	224	150	150	177	177	277	277
**P2S4**	125	125	143	143	162	162	216	216	278	278	179	179	217	217	136	136	265	265	218	218	136	136	255	255	119	119	153	162	189	189	200	200	224	224	150	150	177	177	277	277
**P3S1**	122	122	143	143	159	159	214	214	260	260	189	189	221	221	157	157	295	298	221	221	150	150	284	258	121	121	149	149	188	188	197	197	224	224	140	140	152	152	254	254
**P3S2**	122	122	143	143	159	159	214	214	260	260	189	189	221	221	157	157	295	298	221	221	150	150	281	255	121	121	149	149	186	186	197	197	224	224	140	140	152	152	254	254
**P3S3**	122	122	143	143	159	159	214	214	260	260	189	189	221	221	157	157	295	298	221	221	150	150	281	255	121	121	149	149	186	186	197	197	224	224	140	140	152	152	254	254
**P3S4**	122	122	143	143	159	159	214	214	260	260	189	189	221	221	157	157	295	298	221	221	150	150	281	255	121	121	149	149	186	186	197	197	224	224	140	140	152	152	254	254
**P3S5**	122	122	143	143	159	159	214	214	260	260	189	189	221	221	157	157	295	298	222	222	150	150	284	258	121	121	149	149	188	188	197	197	224	224	140	140	150	150	254	254
**P4S1**	122	122	143	143	159	159	214	214	260	260	179	179	221	221	146	146	271	271	221	222	152	152	284	258	121	121	149	149	188	188	197	197	224	224	140	140	152	152	254	254
**P4S2**	122	122	143	143	159	159	214	214	260	260	179	179	221	221	146	146	271	271	221	221	152	152	284	258	121	121	149	149	188	188	197	197	224	224	140	140	152	152	254	254
**P4S3**	122	122	143	143	159	159	214	214	262	262	179	179	221	221	154	154	298	298	221	221	150	150	284	258	121	121	149	149	188	188	197	197	224	224	140	140	152	152	254	254
**P4S4**	122	122	143	143	159	159	214	214	262	262	179	179	221	221	154	154	271	298	221	221	154	154	284	258	121	121	149	149	188	188	197	197	224	224	140	140	153	153	254	254
**P5S1**	122	122	143	143	159	159	214	214	260	260	189	189	221	221	154	154	271	298	221	221	150	150	281	255	121	121	149	149	186	186	197	197	224	224	140	140	152	152	254	254
**P5S2**	122	122	143	143	159	159	214	214	260	260	189	189	221	221	146	146	271	271	221	221	150	150	281	255	121	121	149	149	186	186	197	197	224	224	140	140	152	152	254	254
**P6S1**	122	122	143	143	162	162	214	214	266	266	189	189	221	221	161	161	283	283	221	221	156	156	279	255	121	121	149	149	186	186	197	197	226	226	140	140	152	152	254	254
**P6S2**	122	122	143	143	162	162	214	214	266	266	189	189	221	221	161	161	283	283	221	221	156	156	281	258	121	121	149	149	188	188	197	197	226	226	140	140	152	152	254	254
**P6S3**	122	122	143	143	162	162	214	214	266	266	189	189	221	221	161	161	283	283	221	221	156	156	279	255	121	121	149	149	188	188	197	197	226	226	140	140	152	152	254	254
**P7S1**	122	122	143	143	162	162	214	214	266	266	189	189	221	221	161	161	283	283	221	221	154	154	279	255	121	121	149	149	186	186	197	197	226	226	140	140	152	152	254	254
**P7S2**	122	122	143	143	162	162	214	214	266	266	189	189	221	221	161	161	283	283	221	221	154	154	279	255	121	121	149	149	186	186	197	197	226	226	140	140	152	152	254	254
**P7S3**	122	122	143	143	162	162	214	214	266	266	189	189	221	221	161	161	283	283	221	221	154	154	279	255	121	121	149	149	185	185	197	197	226	226	140	140	152	152	254	254
**P7S4**	122	122	143	143	162	162	214	214	266	266	189	189	221	221	161	161	283	283	221	221	154	154	279	255	121	121	149	149	185	185	197	197	226	226	140	140	152	152	254	254
**Pram120**	125	125	143	143	162	162	216	216	278	278	179	179	217	217	136	136	265	265	218	218	136	136	255	255	119	119	153	162	188	189	200	200	224	224	150	150	177	177	277	277
**Pram121**	122	122	143	143	162	162	214	214	266	266	189	189	221	221	159	159	283	283	221	221	163	163	281	258	121	121	149	149	188	188	197	197	226	226	140	140	152	152	254	254

Table S4 Dose-response EC50 and standard error summary per sample and for all the tested molecules

	2PEITC			racGR24			+GR24			-GR24			+eGR24			-eGR24		
**sample**	**EC50**	**SE**	**p value**	**EC50**	**SE**	**p value**	**EC50**	**SE**	**p value**	**EC50**	**SE**	**p value**	**EC50**	**SE**	**p value**	**EC50**	**SE**	**p value**
**P1S1**	3.43E-09	1.11E-09	6.12E-03	7.61E-12	2.49E-12	5.12E-03	7.15E-12	2.28E-12	5.95E-03	4.65E-09	1.26E-09	2.17E-03	5.24E-10	1.34E-10	1.38E-03	1.37E-12	4.21E-13	3.71E-03
**P1S2**	3.07E-08	1.26E-08	2.46E-02	9.64E-12	2.85E-12	2.37E-03	1.54E-12	2.65E-13	1.35E-05	7.08E-10	1.50E-09	6.42E-01	3.49E-10	8.83E-11	1.15E-03	2.75E-12	1.03E-12	1.41E-02
**P1S3**	2.97E-08	1.36E-08	3.78E-02	1.26E-11	3.64E-12	1.96E-03	2.66E-12	1.03E-12	1.76E-02	1.28E-09	6.86E-10	7.66E-02	1.57E-10	8.47E-10	8.55E-01	5.63E-12	1.88E-12	9.22E-03
**P1S4**	2.59E-08	4.62E-09	3.23E-05	1.27E-11	4.26E-12	6.26E-03	3.17E-12	1.30E-12	2.43E-02	7.34E-10	1.24E-10	2.07E-05	9.43E-10	1.48E-09	5.32E-01	5.27E-12	1.24E-12	5.51E-04
**P2S1**	3.95E-08	1.81E-08	3.86E-02	1.02E-11	2.53E-12	4.57E-04	1.40E-12	3.03E-13	2.16E-04	1.84E-10	3.71E-10	6.25E-01	9.48E-11	2.87E-10	7.44E-01	5.31E-13	1.61E-13	4.59E-03
**P2S2**	3.28E-08	1.79E-09	1.12E-10	7.24E-12	2.47E-12	8.52E-03	6.56E-12	6.17E-12	3.03E-01	1.14E-09	4.73E-10	2.62E-02	7.71E-10	1.44E-09	5.98E-01	4.41E-12	1.99E-12	3.99E-02
**P2S3**	1.67E-08	4.77E-09	1.71E-03	1.36E-11	6.42E-12	4.40E-02	2.64E-12	7.24E-14	6.69E-03	6.37E-10	7.55E-11	7.57E-08	2.31E-10	4.77E-11	8.67E-05	3.05E-12	1.01E-12	5.94E-03
**P2S4**	1.14E-08	5.08E-09	3.71E-02	2.88E-11	9.00E-12	4.03E-03	8.76E-13	5.56E-13	1.29E-01	6.36E-10	7.78E-09	9.36E-01	2.49E-11	1.19E-10	8.36E-01	6.55E-13	2.89E-13	4.46E-02
**P3S1**	5.67E-08	2.00E-08	1.10E-02	1.90E-09	8.95E-10	4.45E-02	1.86E-10	6.68E-11	1.33E-02	4.76E-09	1.52E-09	4.79E-03	9.05E-10	1.76E-10	4.22E-05	1.84E-12	6.06E-13	5.61E-03
**P3S2**	1.36E-07	1.20E-07	2.94E-06	6.93E-10	1.09E-10	1.49E-06	2.75E-11	9.93E-12	1.42E-02	1.03E-09	2.08E-10	8.01E-05	1.05E-10	2.77E-11	1.63E-03	4.04E-13	3.99E-13	3.23E-01
**P3S3**	6.76E-08	1.18E-08	6.99E-06	1.55E-09	5.02E-10	5.08E-03	8.15E-11	2.74E-11	7.74E-03	8.19E-10	1.03E-09	4.33E-01	1.07E-10	1.10E-10	3.41E-01	1.64E-12	6.77E-13	2.58E-02
**P3S4**	4.18E-08	1.00E-08	3.64E-04	1.03E-09	8.62E-11	1.50E-11	1.14E-11	3.74E-12	1.12E-02	9.32E-10	2.39E-10	9.74E-04	1.35E-11	3.16E-11	6.75E-01	6.23E-13	1.15E-12	5.97E-01
**P3S5**	4.73E-08	8.70E-09	2.99E-05	4.27E-10	2.00E-10	4.28E-02	5.84E-11	2.01E-11	9.74E-03	6.48E-10	3.21E-10	5.77E-02	3.31E-10	2.01E-10	1.18E-01	1.86E-13	1.95E-13	3.52E-01
**P4S1**	8.90E-08	1.20E-08	5.02E-07	1.34E-09	3.53E-10	9.06E-04	9.42E-11	3.57E-11	1.95E-02	3.53E-10	1.41E-10	2.34E-02	1.09E-09	3.84E-10	1.08E-02	1.03E-12	2.03E-13	8.16E-05
**P4S2**	6.15E-07	2.67E-07	3.04E-02	8.79E-10	2.86E-10	5.16E-03	1.47E-10	3.16E-11	6.92E-04	1.14E-09	1.72E-09	5.14E-01	1.47E-09	1.40E-09	3.06E-01	1.82E-12	4.30E-13	9.70E-04
**P4S3**	2.50E-07	6.32E-08	7.23E-04	5.15E-10	1.10E-10	1.04E-04	4.57E-11	5.45E-12	2.99E-07	4.04E-10	1.22E-10	3.25E-03	4.60E-10	7.96E-11	1.80E-05	7.04E-13	2.80E-13	2.21E-02
**P4S4**	2.54E-07	3.80E-08	4.32E-07	6.91E-10	1.28E-10	1.51E-05	4.39E-10	1.48E-10	8.73E-03	9.21E-10	1.56E-10	7.31E-06	4.20E-09	1.45E-09	8.60E-03	2.67E-12	4.06E-13	2.68E-06
**P5S1**	2.92E-09	1.30E-08	2.94E-06	2.29E-10	1.10E-10	4.93E-02	8.81E-11	2.69E-11	4.76E-03	1.72E-09	3.72E-10	1.20E-04	3.38E-10	8.54E-11	6.78E-04	4.03E-13	3.09E-13	2.06E-01
**P5S2**	2.92E-09	1.32E-09	3.72E-02	1.84E-10	7.30E-11	1.90E-02	5.48E-11	2.24E-11	2.40E-02	1.22E-09	4.72E-10	1.73E-02	3.88E-10	7.41E-11	4.64E-05	5.61E-13	7.12E-13	4.46E-01
**P6S1**	6.97E-08	1.79E-08	6.07E-04	9.02E-09	3.59E-09	1.99E-02	2.53E-10	5.00E-11	6.85E-05	5.01E-08	8.05E-09	2.39E-06	3.91E-08	5.83E-09	6.22E-07	4.11E-10	7.40E-11	1.19E-05
**P6S2**	1.83E-08	1.46E-09	1.23E-10	2.18E-09	5.46E-10	6.19E-04	2.17E-10	6.97E-11	7.66E-03	5.24E-08	5.22E-09	4.69E-10	5.49E-08	1.71E-08	3.67E-03	4.16E-10	1.16E-10	1.47E-03
**P6S3**	6.11E-08	2.46E-08	2.05E-02	2.54E-09	6.28E-10	5.93E-04	9.65E-11	2.74E-11	2.28E-03	8.15E-08	4.01E-08	5.39E-02	1.38E-08	1.87E-08	4.69E-01	1.88E-10	5.67E-11	2.98E-03
**P7S1**	4.59E-07	9.19E-08	3.79E-05	1.27E-09	4.12E-10	5.58E-03	1.17E-09	3.93E-10	1.06E-02	9.28E-08	4.80E-09	2.71E-15	2.69E-09	2.14E-08	9.01E-01	3.44E-11	2.31E-10	8.84E-01
**P7S2**	3.91E-07	8.39E-08	1.72E-04	4.11E-10	1.80E-10	3.15E-02	7.21E-10	2.65E-10	1.51E-02	6.99E-07	4.83E-07	1.61E-01	1.57E-07	2.20E-08	4.55E-07	9.87E-10	2.41E-10	4.80E-04
**P7S3**	2.71E-07	3.50E-08	4.18E-08	7.53E-10	2.36E-10	3.89E-03	3.81E-10	1.66E-10	3.69E-02	7.11E-08	1.41E-07	6.19E-01	2.99E-08	2.17E-08	1.91E-01	1.04E-09	2.50E-10	4.78E-04
**P7S4**	1.91E-07	4.79E-08	5.42E-04	1.17E-10	3.97E-11	7.91E-03	9.48E-11	3.11E-11	6.69E-03	5.57E-08	7.86E-09	7.15E-07	9.11E-09	1.62E-09	1.68E-05	1.02E-09	1.17E-10	6.66E-08

Table S5 Prevalence and abundance of *P. ramosa* seed core microbial ASVs in soil samples

Marker gene	ASV identifier and taxonomic identification on SILVA[a] and UNITE[b] databases	Relative prevalence[c] (% of the total prevalence)	Relative abundance[d] (% of the total abundance)	Raw abundance (average number of reads)	Raw abundance in seeds (average number of reads)	Ratio seed/soil
16S	ASV1578_Sphingomonas_unidentified	36.36	0.11	13.18	132.63	10.06
	ASV1971_Rhizobium_unidentified	7.58	0.04	4.67	166.85	35.75
	ASV1998_unidentified	15.15	0.04	5.11	45.90	8.99
	ASV2261_Stenotrophomonas_rhizophila	33.33	0.16	19.17	281.46	14.68
	ASV2265_Stenotrophomonas_unidentified	31.81	0.52	60.74	283.46	4.67
	ASV2268_Stenotrophomonas_chelatiphaga	13.64	0.06	6.50	105.50	16.23
	ASV2876_Paenibacillus_unidentified	6.06	0.01	1.05	102.33	97.88
	ASV2956_Pseudomonas_unidentified	9.10	0.04	5.21	169.51	32.52
	ASV2990_Pseudomonas_unidentified	45.45	0.25	29.03	384.00	13.23
	ASV710_Pedobacter_unidentified	22.73	0.08	9.05	159.83	17.67
ITS	ASV140_Vishniacozyma_victoriae	89.39	1.43	99.18	166.97	1.68
	ASV1817_unidentified	93.94	2.42	167.58	245.27	1.46
	ASV1920_Plectosphaerella_cucumerina	96.97	7.51	519.36	1203.00	2.32
	ASV2459_unidentified	90.91	0.96	66.74	467.49	7.00
	ASV2467_Fusarium_unidentified	100	8.95	618.55	210.63	0.34
	ASV2639_Mycosphaerella_tassiana	93.94	0.84	58.11	240.86	4.15
	ASV2644_Cladosporium_unidentified	98.48	6.29	434.64	2960.45	6.81

[a] SILVA, database of bacterial species (https://www.arb-silva.de/)

[b] UNITE, database of fungal species (https://unite.ut.ee/)

[c] Relative prevalence represents the number of sample in which each seed core ASV is detected expressed as percentage to the total number of sample triplicates

[d] Relative abundance represents the abundance sum of each seed core ASV expressed as percentage to the total abundance in all samples

Table S6 Prevalence and abundance of host related – *P. ramosa* seed core microbial ASVs in soil samples

Marker gene	Host	ASV identifier and taxonomic identification on SILVA[a] and UNITE[b] databases	Relative prevalence[c] (% of the total prevalence)	Relative abundanced (% of the total abundance)	Raw abundance in soil (average number of reads)	Raw abundance in seeds (average number of reads)	Ratio seed/soil
16S	Tobacco	ASV2965_Pseudomonas_unidentified	1.51	< 0.01	0,32	167,33	525,90
	Oilseed rape	ASV3317_Nannocystis_unidentified	1.51	< 0.01	0,39	32,32	144,97
	Tobacco	ASV638_Sphingobacterium_sp. 23D10-4-9	6.06	0.01	1,24	22,58	18,17
	Oilseed rape	ASV668_Sphingobacterium_sp. MIMdw12	Not detected	Not detected	0,00	17,79	Not calculated
	Hemp	ASV718_Pedobacter_unidentified	1.51	< 0.01	0,47	18,86	40,15
ITS	Oilseed rape	ASV1153_unidentified_unidentified	27.27	0.14	9,82	3,81	0,39
		ASV1708_Leptosphaeria_maculans	16.67	0.58	40,02	2,78	0,07

[a] SILVA, database of bacterial species (https://www.arb-silva.de/)

[b] UNITE, database of fungal species (https://unite.ut.ee/)

[c] Relative prevalence represents the number of soil sample in which each seed core ASV is detected expressed as percentage to the total number of soil samples

[d] Relative prevalence represents the number of soil sample in which each seed core ASV is detected expressed as percentage to the total number of soil sample triplicates
